# Actin nucleation at the centrosome controls lymphocyte polarity

**DOI:** 10.1038/ncomms10969

**Published:** 2016-03-18

**Authors:** Dorian Obino, Francesca Farina, Odile Malbec, Pablo J. Sáez, Mathieu Maurin, Jérémie Gaillard, Florent Dingli, Damarys Loew, Alexis Gautreau, Maria-Isabel Yuseff, Laurent Blanchoin, Manuel Théry, Ana-Maria Lennon-Duménil

**Affiliations:** 1 INSERM—U932 Immunité et Cancer, Institut Curie, PSL Research University, 75248 Paris Cedex 05, France; 2 CytoMorpho Lab, Biosciences & Biotechnology Institute of Grenoble, UMR5168, CEA/INRA/CNRS/Université Grenoble-Alpes, Grenoble 38054, France; 3 Laboratoire de Spectrométrie de Masse Protéomique, Institut Curie, PSL Research University, 75248 Paris Cedex 05, France; 4 Ecole Polytechnique, CNRS UMR7654, Palaiseau 91120, France; 5 Departamento de Biologia Celular y Molecular, Pontificia Universidad Catolica de Chile, Santiago 6513677, Chile; 6 CytoMorpho Lab, Hopital Saint Louis, Institut Universitaire d'Hematologie, UMRS1160, CEA/INSERM/Université Paris Diderot, Paris 75010, France

## Abstract

Cell polarity is required for the functional specialization of many cell types including lymphocytes. A hallmark of cell polarity is the reorientation of the centrosome that allows repositioning of organelles and vesicles in an asymmetric fashion. The mechanisms underlying centrosome polarization are not fully understood. Here we found that in resting lymphocytes, centrosome-associated Arp2/3 locally nucleates F-actin, which is needed for centrosome tethering to the nucleus via the LINC complex. Upon lymphocyte activation, Arp2/3 is partially depleted from the centrosome as a result of its recruitment to the immune synapse. This leads to a reduction in F-actin nucleation at the centrosome and thereby allows its detachment from the nucleus and polarization to the synapse. Therefore, F-actin nucleation at the centrosome—regulated by the availability of the Arp2/3 complex—determines its capacity to polarize in response to external stimuli.

## Abstract (web summary)

Cell polarity is marked by re-orientation of the centrosome, but the mechanisms governing centrosome polarization are poorly understood. Here Obino *et al*. show that in lymphocytes centrosome-associated Arp2/3 nucleates actin that tethers the centrosome to the nucleus; activation depletes Arp2/3 from the centrosome and frees it from the nucleus.

## Graphical abstract

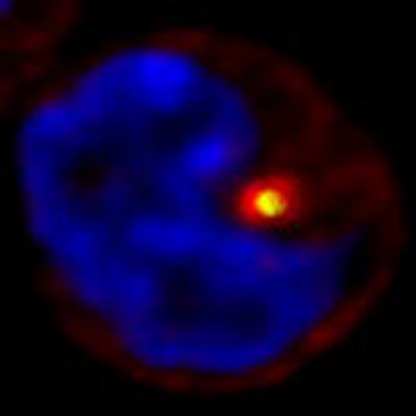

Cell polarity regulates a broad range of biological processes such as cell division, cell fate and cell migration^1^,^2^,^3^. It relies on the organization of the microtubule cytoskeleton, which defines the axis of cell division, as well as the directionality of intracellular trafficking^4^. As the centrosome drives the nucleation and organization of microtubules, this organelle was found to play an essential role in the polarization of a variety of cell types ranging from yeast to specialized cells in multicellular organisms^5^. In lymphocytes, centrosome reorientation to one cell pole was shown to be required for cell migration^6^, asymmetric division^1^,^2^,^7^ and immune synapse formation^8^.

The term immune synapse refers to the zone of tight interaction that forms between lymphocytes and antigen-presenting cells towards which the centrosome polarizes^9^. It is viewed as a signalling platform where both exocytotic and endocytotic events needed for lymphocytes to perform their specific effector function take place^10^. These include the secretion of granules in both cytotoxic lymphocytes and natural killer cells^11^, of cytokine-loaded vesicles in helper T cells^12^,^13^ and of hydrolase-containing lysosomes in B cells^5^,^14^. Hence, centrosome polarization emerges as pivotal in the regulation of immunity, stressing the need to unravel the underlying molecular mechanisms. In that regard, PKC and Cdc42 signalling molecules as well as the microtubule minus-end motor Dynein were shown to regulate centrosome repositioning at the synapse of both B and T lymphocytes^14^,^15^,^16^,^17^,^18^,^19^,^20^. Regarding the actin cytoskeleton, Arp2/3-dependent nucleation of F-actin was shown to be dispensable for centrosome polarization in T lymphocytes, which rather requires the activity of Formins^21^. In general, whether and how centrosome-intrinsic components regulate its ability to polarize remains unexplored.

In this study, we show that Arp2/3-dependent F-actin nucleation at the centrosome of resting lymphocytes links this organelle to the nucleus. Clearance of centrosomal Arp2/3 upon lymphocyte activation promotes centrosome–nucleus separation and subsequent centrosome polarization to the immune synapse. F-actin nucleation at the centrosome therefore determines the ability of this organelle to polarize to one cell pole.

## Results

### Lymphocyte activation modifies the centrosome proteome

**Figure 1:**
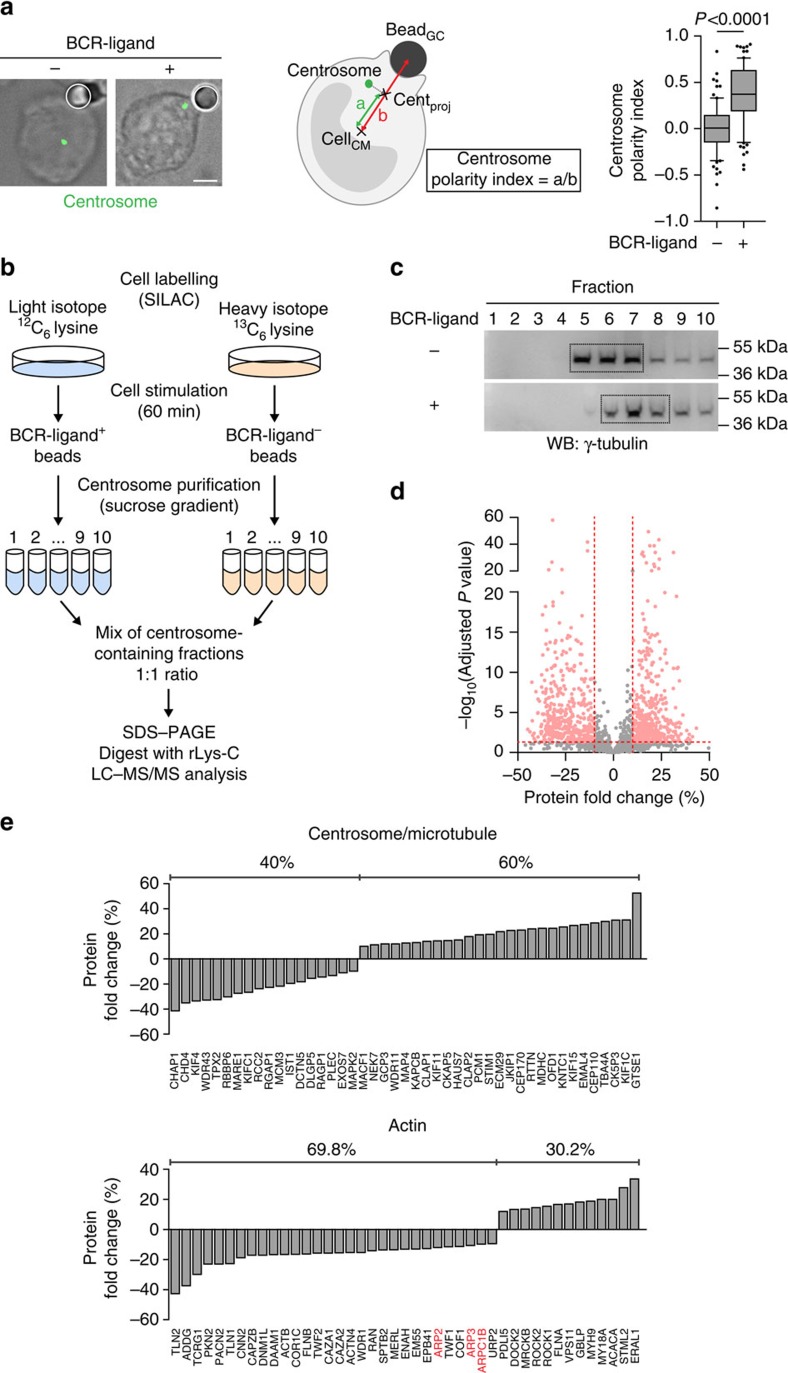
Lymphocyte stimulation modifies the centrosome-associated proteome. (**a**) Left: representative images of non-polarized (BCR-ligand^−^) and polarized (BCR-ligand^+^) B cells. B cells were incubated for 60 min with beads coated with either BCR ligands or with proteins that do not engage the BCR, fixed and the centrosome was stained (γ-tubulin). White circles indicate bead position. Scale bar, 3 μm. Middle: schematics depicting centrosome polarity index measurement. Right: quantification of centrosome polarity index. Data are pooled from three independent experiments with *n*=80 and 85 cells for BCR-ligand^−^ and BCR-ligand^+^, respectively. Unpaired Student's *t*-test was used to determine statistical significance. (**b**) SILAC-based MS workflow used to identify proteins differentially associated with the centrosome of B cells stimulated with either BCR-ligand^−^ or BCR-ligand^+^ beads. (**c**) Western blots highlighting centrosome-containing fractions after centrosome isolation on discontinuous sucrose gradient. Immunoblots are representative of three independent experiments. (**d**) Volcano plot showing the 835 proteins considered for further analysis (light red) among the total of the 1,600 quantified proteins. Horizontal red line represents the threshold for statistical significance (adjusted *P* value ≤0.05). Vertical red lines represent the biological threshold used to select proteins (−10% and +10% of protein fold change). (**e**) Protein fold change (%) for each of the 45 proteins belonging to the ‘centrosome/microtubule' subgroup (top) and the 43 belonging to the ‘actin' one (bottom).

We aimed at investigating the role of centrosome-associated proteins in cell polarity by using B lymphocytes as a model. Centrosome polarization in these cells can be triggered by engaging their membrane antigen receptor—the B-cell antigen receptor (BCR)—with surface-tethered ligands coated on latex beads (Fig. 1a), planar surfaces or cells^14^,^17^,^22^. We hypothesized that changes in the composition of centrosome-associated proteins between non-polarized and polarized cells might reveal valuable candidates to be involved in this process. A stable isotope labelling by amino acids in cell culture (SILAC)^23^-based quantitative proteomic approach was therefore developed to identify proteins differentially associated with the centrosome of non-polarized and polarized B cells. For this, B cells were grown in cultures containing lysine labelled with light or heavy carbon isotopes and incubated for 60 min with BCR-ligand^+^ or BCR-ligand^−^ beads, respectively (Fig. 1b). Cells were lysed, centrosomes were isolated on sucrose gradient and the three main γ-tubulin-containing fractions were pooled for each sample (Fig. 1c). Resulting pools were mixed 1:1 to be separated by SDS–polyacrylamide gel electrophoresis (SDS–PAGE) followed by reverse-phase liquid chromatography and analysed by high-resolution mass spectrometry (LC–MS/MS) (Fig. 1b). This led to the quantification of 1,600 proteins (false discovery rate (FDR) of 1%, number of peptides used ≥3; Fig. 1d) among which 835 were differentially associated with the centrosome of activated lymphocytes (absolute fold change ≥10% and adjusted *P* value of quantification ≤0.05; Fig. 1d, light red). To identify key networks, genome ontology (GO) term enrichment was performed on these 835 proteins. As expected, this analysis highlighted components of the microtubule-organizing centre (enrichment factor: 1.9; *P* value=3.56 × 10^−05^) and the cytoskeleton (enrichment factor: 1.8; *P* value=2.65 × 10^−11^) as two major groups of proteins enriched in centrosome preparations (Supplementary Table 1). More surprisingly, zooming on proteins belonging to the GO term ‘Cytoskeleton' showed that while microtubule-related components were either increased or decreased in polarized cells, the majority of actin cytoskeleton components were reduced (69.8%; Fig. 1e and Supplementary Table 2). Noticeably, this particularly applied to three subunits of the branched actin-nucleating complex Arp2/3 (ref. 24; 10 and 12% decrease; Fig. 1e, red; and Supplementary Tables 3–5). Immunoblot analysis showed an even more pronounced reduction of the Arp2/3 subunit Arp2 in centrosomal fractions from activated lymphocytes (Supplementary Fig. 1a). No reduction in the total amount of Arp2 was observed between both conditions (Supplementary Fig. 1b). We conclude that BCR engagement induces multiple changes in the centrosome proteome including a significant reduction in the pool of associated Arp2/3. Although the presence of this complex at the centrosome had been described in the past^25^, whether it regulates centrosome function remains unclear. We therefore focused our analysis on exploring the putative role of Arp2/3 reduction at the centrosome of activated lymphocytes in the polarization of this organelle.

### Reduced centrosomal Arp2/3 in activated lymphocytes

**Figure 2:**
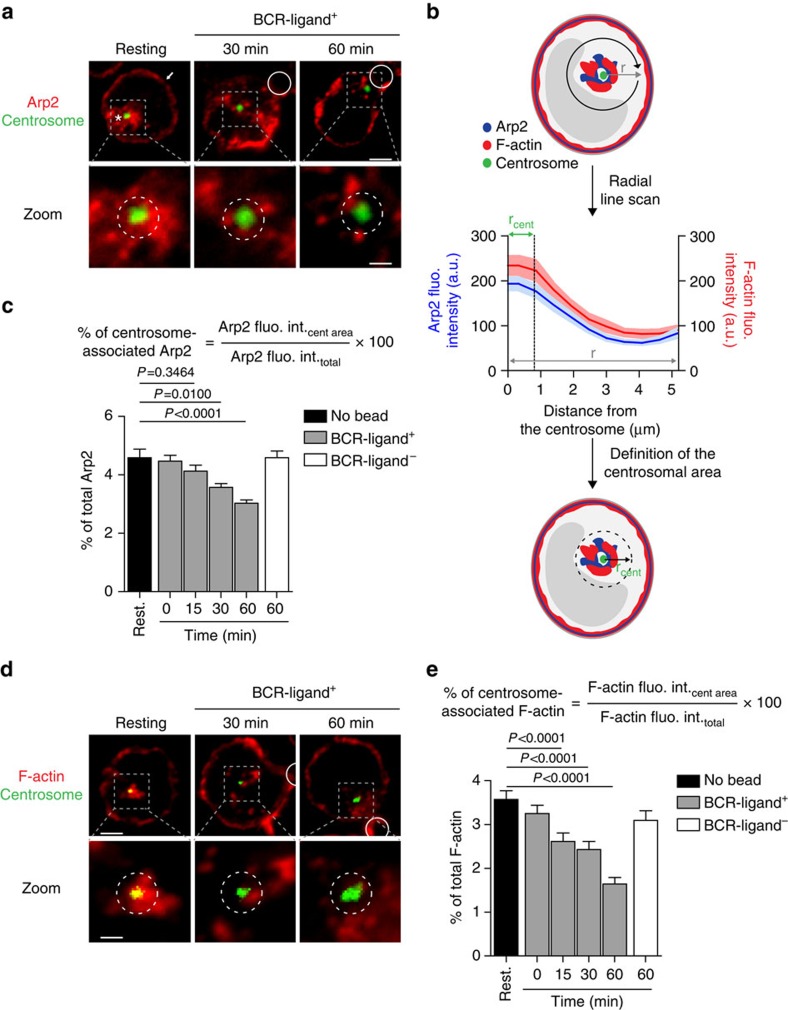
Decreased association of Arp2/3 and F-actin with centrosomes of BCR-stimulated lymphocytes. (**a**,**d**) Representative images of B cells under resting conditions or stimulated with BCR-ligand^+^ beads for indicated time, fixed and co-stained for Arp2 (white arrow: cortical pool; *: centrosomal pool) (**a**) or F-actin (phalloidin) (**d**) and the centrosome (α-tubulin). White circles indicate bead position. Dashed grey squares indicate the centrosomal region magnified below each image. Dashed circles on bottom panel highlight the centrosomal area used for quantification. Scale bars, top: 3 μm; bottom: 1 μm. (**b**) Schematics depicting the pipeline used to quantify centrosome-associated Arp2 and F-actin. (**c**,**e**) Quantification of centrosome-associated Arp2 (**c**) and F-actin (**e**) from cells shown in **a** and **d**, respectively. Data are pooled from three independent experiments with (**c**) *n*=67, 62, 64, 72, 61 and 69 cells, and (**e**) *n*=54, 63, 64, 62, 59 and 64 cells from left to right, respectively. fluo. int., fluorescence intensity. cent, centrosome. Rest., Resting.

We next asked whether reduction of Arp2/3 at the centrosome was equally observed in intact lymphocytes. Immunofluorescence analysis revealed the presence of two pools of Arp2/3 in resting B cells: a cortical pool (Fig. 2a, white arrow) and a cytosolic pool that surrounded the centrosome (Fig. 2a, white star). To accurately quantify this centrosome-associated pool of Arp2/3, we computed a radial line scan of Arp2 fluorescence intensity from the centrosome of resting lymphocytes and, based on this result, we defined a ‘centrosomal area' (Fig. 2b). The amount of Arp2/3 in this centrosomal area was then quantified at different time points after BCR engagement. In agreement with our proteomic and immunoblot data, we found that this centrosome-associated pool of Arp2/3 gradually decreased in time upon lymphocyte stimulation with BCR-ligand^+^ beads (Fig. 2a,c). No reduction in the total amount of Arp2/3 was found (Supplementary Fig. 2a). Similarly, in resting B lymphocytes, we observed the presence of a pool of F-actin in close vicinity of the centrosome (Fig. 2d), which co-localized with Arp2/3 (Supplementary Fig. 2b). In contrast, in lymphocytes incubated for 30 min with BCR-ligand^+^ beads, F-actin was observed as patches dispersed in the cytosol rather than gathered around the centrosome (Fig. 2d). After 60 min of stimulation, the centrosome polarized to the cell–bead interface and was therefore found in proximity to the cortical F-actin pool. Nonetheless, the pool of centrosome-associated F-actin was decreased in these cells (Fig. 2b,d,e). No reduction in the total amount of F-actin was observed (Supplementary Fig. 2c). Of note, because methanol fixation required for γ-tubulin staining is not compatible with phalloidin labelling, the centrosome was stained with antibodies directed against α-tubulin, and images were processed (fluorescence intensity threshold) to visualize the centrosome but not the microtubules (Supplementary Fig. 2d). Altogether these results show that resting B cells display a pool of Arp2/3 and F-actin at their centrosome that decreases while this organelle polarizes to the immune synapse.

### Reduced F-actin nucleation at activated lymphocyte centrosomes

**Figure 3:**
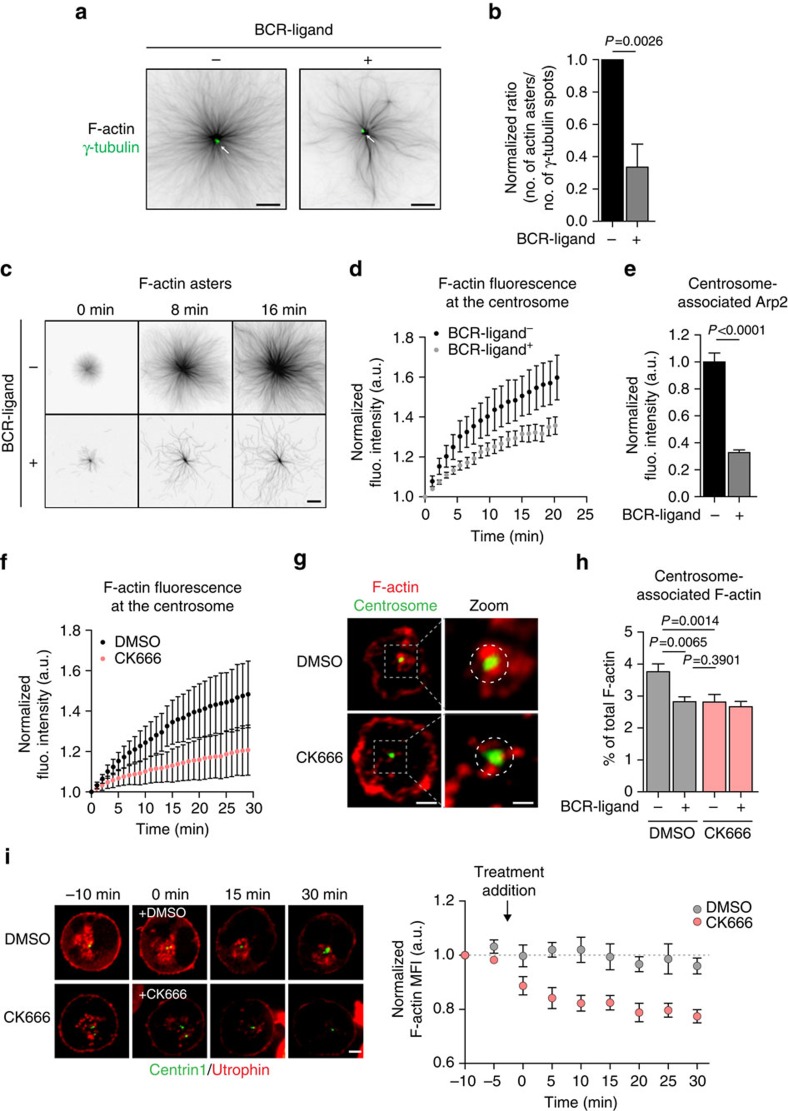
F-actin nucleation by centrosomes is downregulated upon BCR stimulation. (**a**) Representative images of actin asters nucleated from isolated centrosomes (white arrow). Scale bar, 8 μm. (**b**) Actin nucleation efficiency was calculated as the ratio of the number of actin asters divided by the number of γ-tubulin spots (*>*200 actin asters and *>*450 γ-tubulin spots per condition pooled from four independent experiments). (**c**) Sequential images of F-actin assembly by centrosomes isolated from B cells stimulated with indicated beads. Scale bar, 5 μm. (**d**) Quantification of F-actin nucleation activity (*n=*14 and 12 actin asters per condition; data are representative of four independent experiments) (**e**) Quantification of Arp2 fluorescence (fluo.) intensity associated with purified centrosomes (*n*=100 and 190 centrosomes per condition pooled from two independent experiments). (**f**) Quantification of actin nucleation activity of centrosomes purified from resting lymphocytes in the presence of CK666 or dimethylsulfoxide (DMSO; *n*=12 and 22 actin asters, respectively; data are representative of two independent experiments). (**g**) Representative images of resting B cells treated with DMSO or CK666 for 60 min, fixed and co-stained for F-actin (phalloidin) and the centrosome (α-tubulin). Dashed grey squares indicate the region magnified on the right panel. Dashed circles on the right panel highlight the centrosomal area used for quantification. Scale bars, left: 3 μm; right: 1 μm. (**h**) Quantification of centrosome-associated F-actin of B cells pretreated for 30 min with DMSO or CK666 before being stimulated with indicated beads for 60 min in presence of the drug (>60 cells per condition pooled from three independent experiments). (**i**) Left: sequential images of resting B cells co-transfected with the centrosomal marker eGFP–Centrin1 and the F-actin probe Utrophin-RFP imaged by time-lapse spinning disk microscopy and treated with either DMSO or CK666 (between *t*_−5 min_ and *t*_0 min_). Scale bar, 3 μm. Right: quantification of centrosome-associated F-actin over time. Centrosomal F-actin mean fluorescence intensity (MFI) was normalized with respect to initial intensity (*t*_−10 min_) for each cell. Data are pooled from two independent experiments and graph shows the mean±s.e.m. of *n*=8 and 10 cells for DMSO and CK666, respectively. *P* values determined by Mann–Whitney test.

Having recently shown that centrosomes possess an intrinsic actin-nucleating activity in various cell types including T lymphocytes^26^, we investigated whether the distinct amounts of centrosome-associated F-actin observed in resting and BCR-stimulated B lymphocytes reflected different actin nucleation capacities. For this, centrosomes purified from resting and activated B lymphocytes were compared for their ability to nucleate actin filaments *in vitro*. Strikingly, we observed that both centrosome preparations assembled actin asters from γ-tubulin spots (Fig. 3a), indicating that centrosomes possess an intrinsic actin nucleation capacity. In agreement with our hypothesis, actin nucleation by centrosomes purified from BCR-stimulated cells was strongly diminished as compared with centrosomes purified from resting lymphocytes. Indeed, both the number of actin asters and the actin fluorescence intensity at the aster centre were significantly decreased when using centrosomes from activated cells (Fig. 3b–d and Supplementary Movie 1). Importantly, centrosome integrity was not affected in preparations from activated lymphocytes, as shown by their ability to nucleate microtubules (Supplementary Fig. 3a). Consistent with these results and with our proteomic and immunofluorescence data, the amount of Arp2/3 associated with centrosomes purified from BCR-stimulated lymphocytes was also found to be strongly decreased as compared with centrosomes of resting B cells (Fig. 3e).

The involvement of the Arp2/3 complex in F-actin nucleation by centrosomes purified from resting lymphocytes was confirmed by using the CK666 Arp2/3 inhibitor^27^, which significantly reduced F-actin assembly (Fig. 3f). On the same line, treatment of resting B cells with CK666 decreased the amount of centrosome-associated F-actin to the levels observed in BCR-stimulated lymphocytes (Fig. 3g,h). Equivalent results were obtained when silencing Arp2/3 with two different siRNA (Supplementary Fig. 3b,c) or when using the Utrophin-red fluorescent protein (RFP) probe to label F-actin in live-imaging experiments: addition of CK666 resulted in decreased centrosomal F-actin in non-activated lymphocytes (Fig. 3i and Supplementary Movie 2). Interestingly, a significant reduction in the fraction of centrosome-associated Arp2/3 was also observed in CK666-treated resting B cells (Supplementary Fig. 3d), suggesting that Arp2/3 activity is required for its localization at the centrosome and/or that the presence of branched actin at the centrosome might help locally maintaining the complex. Of note, formin inhibition did not decrease F-actin nucleation at the centrosome, indicating that it most likely did not play a direct role in this process. Interestingly, formin inhibition even increased the amount of F-actin at the centrosome, what might result from the recently reported competition between Arp2/3 and formins^28^ (Supplementary Fig. 3e,f). We conclude that lymphocyte centrosomes nucleate F-actin in an Arp2/3-dependent manner and that this property of centrosomes is downregulated upon lymphocyte activation as a result of Arp2/3 local depletion.

### Arp2/3 recruitment to the immune synapse

**Figure 4:**
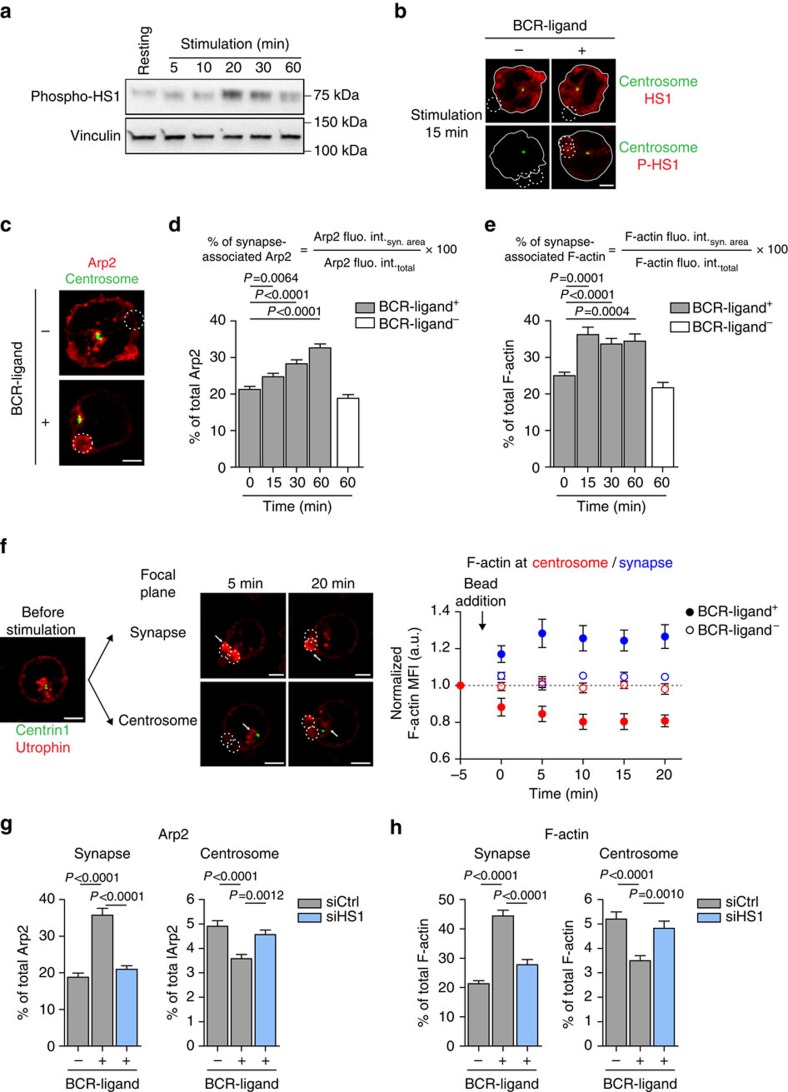
Depletion of Arp2/3 from the centrosome results from its HS1-dependent recruitment at the immune synapse. (**a**) Western blot showing the phosphorylation of HS1 during the course of B-cell stimulation. Representative of two independent experiments. (**b**) Representative images of B cells stimulated with indicated beads for 15 min, fixed and co-stained for total HS1 (top) or phosphorylated HS1 (bottom) and the centrosome (α-tubulin). Dashed white circles indicate bead position. Scale bar, 3 μm. Images are representative of two independent experiments. (**c**) Representative images of B cells stimulated with indicated beads for 60 min, fixed and co-stained for Arp2 and the centrosome (α-tubulin). Dashed white circles indicate bead position. Scale bar, 3 μm. Representative of three independent experiments. (**d**,**e**) Quantification of synapse-associated Arp2 (**d**) and F-actin (**e**). (**d**) *n*=71, 64, 68, 72 and 69 cells and (**e**) *n*=55, 60, 66, 59 and 57 cells from left to right, pooled from three independent experiments. (**f**) Left: sequential images of B cells co-transfected with the centrosomal marker eGFP–Centrin1 and the F-actin probe Utrophin-RFP stimulated with BCR-ligand^+^ beads and imaged by time-lapse spinning disk microscopy. White arrows indicate F-actin clearance from the centrosome (bottom) and its concomitant accumulation at the immune synapse (top) during the course of B-cell stimulation. Dashed white circles indicate bead position. Scale bar, 3 μm. Right: quantification of synapse- (blue) and centrosome- (red) associated F-actin of B cells stimulated with indicated beads. F-actin mean fluorescence intensity (MFI) was normalized with respect to initial intensity (*t*_−5 min_) for each cell. Data are pooled from two independent experiments and graph shows the mean±s.e.m. of *n*=7 cells per condition. (**g**,**h**) Quantification of Arp2 (**g**) and F-actin (**h**) associated with the synapse (left) and the centrosome (right) in control and HS1-silenced B cells stimulated for 60 min with indicated beads. Data are pooled from two (**g**) and three (**h**) independent experiments with (**g**) synapse: *n*=51, 46 and 53 cells; centrosome: *n*=51, 52 and 47 cells; and (**h**) synapse: *n*=72, 74 and 66 cells; centrosome: *n*=73, 72 and 67 cells from left to right. *P* values determined by Mann–Whitney test. fluo. int., fluorescence intensity. syn, synapse.

We next searched for the molecular mechanisms responsible for this partial depletion of Arp2/3 from the centrosome of BCR-stimulated lymphocytes. It was shown that the Cortactin homologue haematopoietic lineage cell-specific protein (HS1), which is predominantly expressed in haematopoietic cells^29^, recruits Arp2/3 to the BCR signalosome upon antigenic stimulation^30^. Consistently, we observed that BCR engagement with BCR-ligand^+^ beads induced HS1 phosphorylation and accumulation at the cell–bead interface (Fig. 4a,b). We therefore hypothesized that phospho-HS1-dependent recruitment of Arp2/3 at the immune synapse might lead to its partial depletion from the centrosome and thereby to a local decrease in F-actin nucleation. Consistent with this hypothesis, we found that the gradual decrease in the pool of centrosome-associated Arp2/3 and F-actin was concomitant to the accumulation of both proteins at the cell–bead interface (Fig. 4c–e and Supplementary Fig. 4a). This was also observed for F-actin in time-lapse imaging experiments: upon BCR stimulation, F-actin gradually decreased at the centrosome but progressively increased at the synapse (Fig. 4f and Supplementary Movie 3 and 4). Noticeably, both the decrease of Arp2/3 and F-actin at the centrosome and their increase at the synapse were severely impaired when silencing HS1 (Fig. 4g,h and Supplementary Fig. 4b). No reduction in centrosome-associated F-actin, nor in the total amount of F-actin was observed between control and HS1-silenced lymphocytes (Supplementary Fig. 4c,d). Hence, HS1-dependent recruitment of Arp2/3 at the cell–bead interface is associated with its partial depletion from the centrosome, thus decreasing the actin nucleation capacity of this organelle.

### Centrosomal Arp2/3 and actin impair centrosome polarization

**Figure 5:**
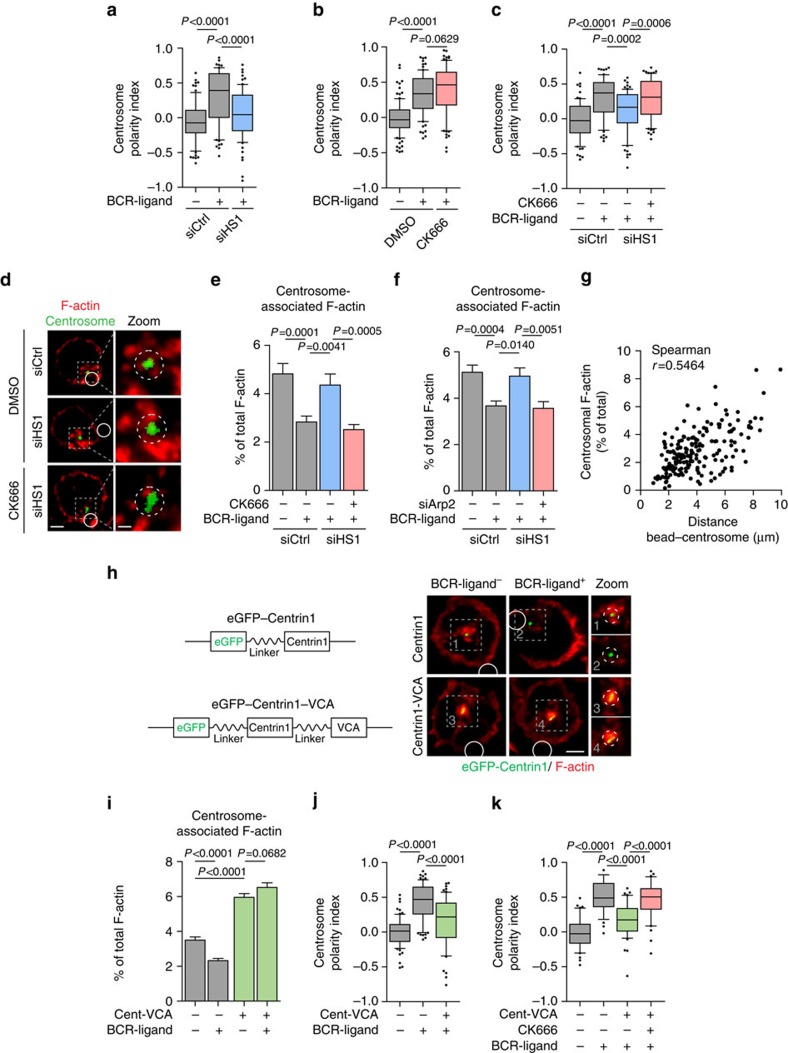
Downregulation of centrosomal Arp2/3-dependent F-actin nucleation is required for centrosome polarization to the immune synapse. (**a**–**c**) Quantification of centrosome polarity index of control and HS1-silenced (**a**), CK666-treated (**b**) or HS1-silenced and CK666-treated (**c**) B cells stimulated with indicated beads for 60 min. (**a**) *n*=77, 71 and 75 cells; (**b**) *n*=103, 80 and 87 cells and (**c**) *n*=77, 69, 72 and 75 cells from left to right, pooled from three independent experiments. (**d**) Representative images of control and HS1-silenced B cells treated with dimethylsulfoxide (DMSO) or CK666, stimulated with BCR-ligand^+^ beads for 60 min, fixed and co-stained for F-actin (phalloidin) and the centrosome (α-tubulin). Scale bars, left: 3 μm; right: 0.9 μm. (**e**) Quantification of centrosome-associated F-actin from cells shown in **d** (*n*=41, 47, 38 and 45 cells from left to right, pooled from two independent experiments). (**f**) Quantification of centrosomal F-actin of control, HS1- and HS1 plus Arp2-silenced B cells stimulated for 60 min with indicated beads (*n*=58, 65, 65 and 63 cells from left to right, pooled from three independent experiments). (**g**) Correlation analysis of centrosome-associated F-actin and the bead–centrosome distance (*n*=185 cells). Spearman correlation test, *P*<0,0001. (**h**) Left: schematics depicting the construct used to over-activate the Arp2/3 complex at the centrosome (bottom). Right: representative images of control and eGFP–Centrin1–VCA-expressing B cells, stimulated with indicated beads for 60 min, fixed and co-stained for F-actin (phalloidin) and the centrosome (GFP). Scale bar, 3 μm. (**d**,**h**) White circles indicate bead position. Dashed grey squares indicate the region magnified on the right. Dashed circles on magnifications highlight the centrosomal area used for quantification. (**i**,**j**) Quantification of centrosomal F-actin (**i**) and centrosome polarity index (**j**) of cells shown in **h**. (**i**) *n*=74, 66, 68 and 64 cells and (**j**) *n*=75, 71 and 64 cells from left to right, pooled from three independent experiments. (**k**) Quantification of centrosome polarity index of control and eGFP–Centrin1–VCA-expressing B cells, treated or not with CK666 and stimulated with indicated beads for 60 min (*n*=41, 39, 42 and 42 cells from left to right, pooled from two independent experiments). *P* values determined by Mann–Whitney test. Cent-VCA, eGFP-centrin1-VCA.

HS1-silenced lymphocytes that maintained high levels of centrosomal Arp2/3 and F-actin upon BCR stimulation were next used to investigate whether Arp2/3 and F-actin depletion from the centrosome regulates the ability of this organelle to polarize to the immune synapse. We found that most HS1 knockdown B cells did not reposition their centrosome at the cell–bead interface (Fig. 5a). However, because these cells displayed not only more Arp2/3 and F-actin at the centrosome but also less Arp2/3 and F-actin at the synapse as compared with control cells, we could not exclude that impaired centrosome polarization resulted from decreased Arp2/3 and F-actin at the synapse. To address this question, we investigated the effect of Arp2/3 inhibition on centrosome polarity. We found that both Arp2/3 silencing and inhibition with CK666 had no impact on centrosome polarization to the synapse (Fig. 5b and Supplementary Fig. 5a) and, even more importantly, rescued the non-polarized phenotype of HS1-silenced activated lymphocytes (Fig. 5c and Supplementary Fig. 5b,c). Of note, Arp2/3 inhibition in these cells reduced the centrosomal pool of F-actin (Fig. 5d–f) but had no significant effect on the amounts of synapse-associated F-actin (Supplementary Fig. 5d). CK666 treatment had no impact on BCR signalling (Supplementary Fig. 5e,f). These results suggest that the centrosomal pool of Arp2/3 and F-actin prevents centrosome polarization while its synaptic counterpart is not required for this process. In support of this conclusion, a significant correlation was found between the levels of centrosomal F-actin and the distance between this organelle and the bead geometrical centre (Fig. 5g). Hence, HS1-dependent recruitment of Arp2/3 at the synapse partially depletes this complex from the centrosome, leading to a local reduction in F-actin that is needed for centrosome polarization to the synapse.

We next sought for a strategy to directly assess whether actin nucleation by Arp2/3 at the centrosome prevents the polarization of this organelle to the immune synapse. Interestingly, WASH, an actin nucleation-promoting factor that activates Arp2/3 through its VCA (verprolin homology or WH2-connector-acidic) domain, was shown to associate to the centrosome^31^. In agreement, in resting lymphocytes, we observed WASH as discrete punctuated structures mainly gathered around the centrosome (Supplementary Fig. 5g). In addition, in resting cells, WASH silencing decreased the amount of F-actin at the centrosome to the levels observed in activated cells (Supplementary Fig. 5h–j), indicating that it participates to local Arp2/3 activation. We therefore reasoned that targeting the WASH VCA domain to the centrosome would result in the exacerbation of local Arp2/3 activity and F-actin nucleation. Accordingly, expression of the eGFP–Centrin1–VCA fusion protein strongly increased the amount of F-actin at the centrosome (Fig. 5h,i). More importantly, expression of the eGFP–Centrin1–VCA fusion protein compromised the ability of the centrosome to polarize to the immune synapse (Fig. 5j). As observed for Arp2/3 inhibition or silencing, WASH silencing had no impact on centrosome polarity (Supplementary Fig. 5k). Consistently, centrosome polarization in eGFP–Centrin1–VCA-expressing cells was rescued by inhibiting Arp2/3 activity (Fig. 5k). These data strongly support a model where F-actin nucleation at the centrosome prevents its translocation to the synapse and must therefore be downregulated upon lymphocyte activation.

### Centrosomal F-actin tethers the centrosome to the nucleus

**Figure 6:**
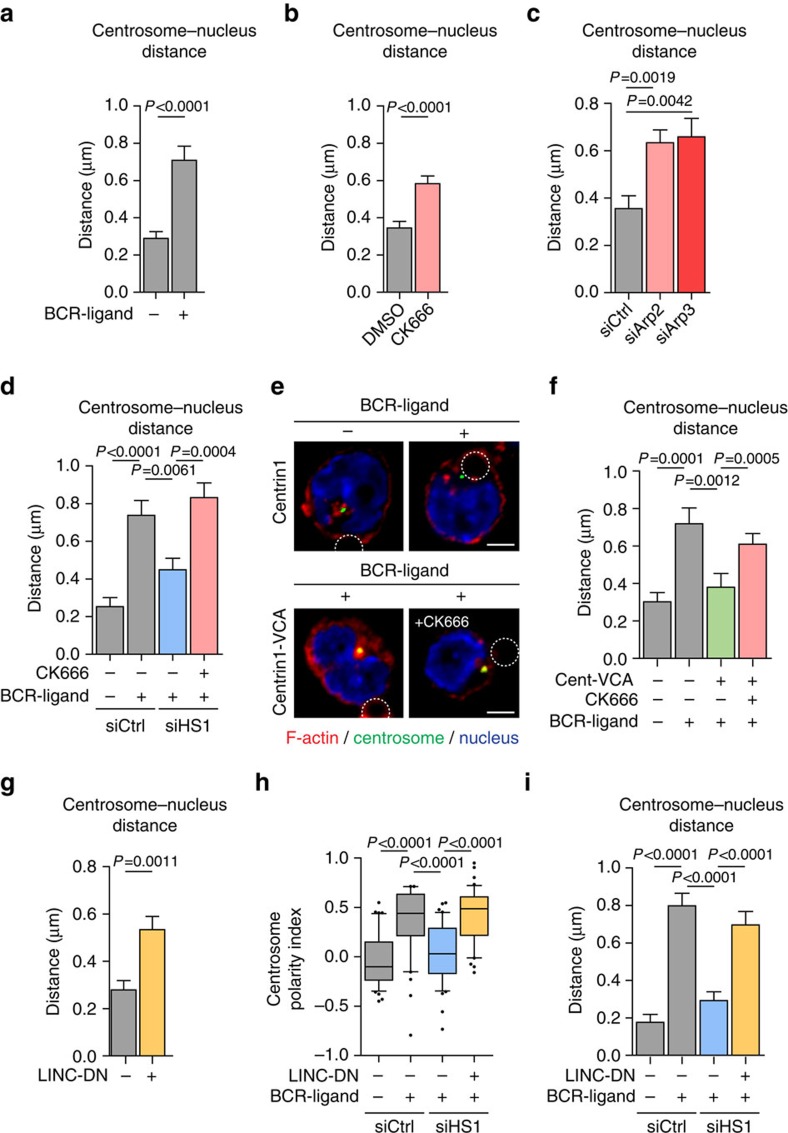
Centrosomal Arp2/3-mediated F-actin nucleation links the centrosome to the nucleus through the LINC complex. (**a**–**d**) The shorter distance in three dimensions between the centrosome and the edge of the nucleus was measured in: (**a**) B cells stimulated with either BCR-ligand^−^ or BCR-ligand^+^ beads for 60 min; (**b**) resting B cells treated with dimethylsulfoxide (DMSO) or CK666 for 60 min; (**c**) control, Arp2- and Arp3-silenced resting B cells; and (**d**) control and HS1-silenced B cells, treated with either DMSO or CK666, and stimulated with indicated beads for 60 min. Data are pooled from two (**c**,**d**) and three (**a**,**b**) independent experiments with (**a**) *n=*90 cells per condition; (**b**) *n=*93 and 78 cells for DMSO and CK666, respectively; (**c**) *n=*43, 35 and 31 cells; and (**d**) *n=*56, 53, 52 and 54 cells from left to right. (**e**) Representative images of B cells over-expressing the eGFP–Centrin1 protein or the eGFP–Centrin1–VCA fusion protein treated or not with CK666, stimulated with indicated beads for 60 min, fixed and co-stained for F-actin (phalloidin), the centrosome (GFP) and the nucleus (DAPI). Dashed circles indicate bead position. Scale bar, 3 μm. (**f**) Quantification of the distance between the centrosome and the nucleus edge from cells shown in **e**. Data are pooled from two independent experiments with *n=*41, 39, 42 and 43 cells from left to right. (**g**) Quantification of the distance between the nucleus edge and the centrosome in resting B cells over-expressing or not the LINC-DN construct. Data are pooled from three independent experiments with *n=*64 cells per condition. (**h**,**i**) Quantification of centrosome polarity index (**h**) and centrosome–nucleus distance (**i**) of control and HS1-silenced B cells, over-expressing or not the LINC-DN construct and stimulated for 60 min with indicated beads. Data are pooled from two independent experiments with (**h**) *n=*41, 40, 42 and 42 cells and (**i**) *n=*41, 44, 41 and 45 cells from left to right. *P* values determined by Mann–Whitney test. DAPI, 4′,6-diamidino-2-phenyindole.

We next searched for the cellular basis of the negative impact of Arp2/3-dependent actin nucleation at the centrosome on its ability to polarize. In view of a recent work indicating that F-actin controls centrosome positioning by inducing the retrograde transport of the nucleus in polarized fibroblasts^32^, we postulated that actin nucleation at the centrosome might regulate its physical interaction with the nucleus^33^. To test this hypothesis, we measured the distance between both organelles in lymphocytes that exhibited different levels of centrosomal F-actin. We found that reduction of centrosomal F-actin upon BCR engagement not only stimulated centrosome polarization but was also accompanied by an increase in the distance between the nucleus and this organelle (Fig. 6a). Strikingly, such increase was equally observed when depleting or inhibiting Arp2/3 in non-stimulated cells (Fig. 6b,c), indicating that the mere reduction of centrosomal F-actin is sufficient to induce its physical separation from the nucleus. Similarly, the distance between the centrosome and the nucleus of resting WASH-silenced cells, whose centrosome has low levels of centrosomal F-actin, was also increased (Supplementary Fig. 6a). In contrast, in activated HS1-silenced lymphocytes that maintained high levels of F-actin at their centrosome, the centrosome–nucleus distance was as short as in non-stimulated cells (Fig. 6d). This result equally applied to lymphocytes expressing the eGFP–Centrin1–VCA construct, which displayed increased centrosomal F-actin and impaired centrosome polarization (Fig. 6e,f). However, centrosome–nucleus separation was rescued in these cells by reducing centrosomal F-actin with CK666 or by silencing Arp2/3 (Fig. 6d–f and Supplementary Fig. 6b). These results strongly suggest that the pool of F-actin at the centrosome maintains it in close proximity to the nucleus and must therefore be depleted for these two organelles to physically separate. Important molecules involved in the physical association of the centrosome to the nucleus are components of the linker of nucleoskeleton and cytoskeleton (LINC) complex. This complex includes Nesprin proteins that bind both the microtubule and actin cytoskeleton networks. We therefore investigated whether detachment of the centrosome from the nucleus as a result of LINC complex disruption might rescue centrosome polarity. Over-expression of a dominant-negative mutant of Nesprin-2 that does not bind F-actin^32^ was sufficient to increase the distance between the centrosome and the nucleus in resting cells without affecting the amounts of centrosome-associated F-actin (Fig. 6g and Supplementary Fig. 6c). More importantly, expression of this dominant-negative version of the LINC complex rescued both centrosome polarization and centrosome separation from the nucleus in HS1-silenced activated lymphocytes despite their high levels of centrosomal F-actin (Fig. 6h,i and Supplementary Fig. 6d,e). Hence, the need to deplete F-actin at the centrosome to detach it from the nucleus and allow its polarization to the immune synapse can be bypassed by disrupting the LINC complex. These results strongly suggest that F-actin nucleation at the centrosome is required for its physical association to the nucleus by the LINC complex. Altogether, these data further provide a putative mechanism for the need to deplete centrosomal Arp2/3 and F-actin to allow centrosome translocation to the immune synapse.

## Discussion

We here found that BCR engagement with immobilized antigens induces the accumulation of the Cortactin-like protein HS1 at the immune synapse, which recruits Arp2/3, promoting the local enrichment of F-actin. Of note, although inhibition of Arp2/3 does not impair centrosome polarization, its activity is nonetheless required for B lymphocytes to process and present BCR-internalized antigens to T lymphocytes (Supplementary Fig. 6f), suggesting a function for this complex at the B-cell synapse. Recruitment of Arp2/3 at the synapse leads to its partial depletion from the centrosome, thereby reducing the pool of centrosome-nucleated F-actin. The centrosome would then be free to physically separate from the nucleus and move towards the immune synapse. Importantly, we found that an intact LINC complex is required for centrosomal F-actin to maintain centrosome attachment to the nucleus. Interestingly, although the molecular players that allow interaction between the LINC complex and the nucleoskeleton were described, the one that link this complex to the centrosome remained unclear^33^. Our results suggesting that F-actin nucleation at the centrosome might be a key player in this process therefore brings an interesting new piece to this puzzle.

Our findings strongly suggest that F-actin nucleation at the centrosome must decrease for this organelle to acquire a polarized localization. This might result, at least in part, from the role of centrosomal F-actin in retaining the centrosome in vicinity of the nucleus^33^. The idea that the actin cytoskeleton links the centrosome to the nucleus was initially proposed in the eighties based on observations showing that nucleus purification or cell enucleation required the addition of F-actin-depolymerizing drugs^34^,^35^. However, the precise nature and origin of this actin network remain unclear. Different F-actin structures had been reported in association to the centrosome and/or the nucleus. This includes ‘actin clouds' that position centrosomes and mitotic spindles^36^ and resemble the F-actin structures we observed at the centrosome of B lymphocytes. In addition, the nucleus of migrating fibroblasts associates to a ‘perinuclear actin cap'^37^ or to ‘linear actin arrays' referred to as TAN lines, which regulate nucleus retrograde transport and centrosome polarity through the LINC complex^32^,^38^. Although we did not observe TAN lines in B lymphocytes, what might be due to their non-adherent properties, whether and how Arp2/3-dependent F-actin nucleation at the centrosome contributes to the formation and/or function of these actin structures will be an important point to investigate in the future.

Of note, we do not exclude that actin depletion at the centrosome may control additional processes required for efficient centrosome polarization than centrosome–nucleus attachment. In particular, reduction of F-actin nucleation at the centrosome might induce local changes to favour its microtubule-dependent translocation to the synapse. For example, it may facilitate Dynein recruitment and/or local centrosome docking at the immune synapse. Consistent with these hypotheses, we and others have shown that Dynein is indeed required for centrosome reorientation to the synapse in both B and T lymphocytes^15^,^17^,^19^,^20^. Further work is needed to unravel the role played by centrosome-nucleated F-actin in the biology of resting lymphocytes, as well as to fully understand how depletion of this F-actin pool facilitates centrosome polarity.

Our data suggest that the centrosome and the immune synapse compete for Arp2/3. Although we cannot formally exclude that there is a direct exchange between centrosomal and synaptic Arp2/3, we think that this is unlikely in view of data showing that most Arp2/3 resides in the cytosol^39^. We therefore propose that both the centrosome and the synapse are rather competing for the cytosolic pool of Arp2/3.

Anyhow, our data strongly suggest that there is an effective competition between distinct subcellular locations for this actin-nucleating complex. As a result of this competition, cells would respond to extracellular stimuli by favouring one biological process over another, what might provide a simple mechanism for cells to coordinate them in time and space. In addition, the existence of a competition for cytosolic Arp2/3 implies that the pool of centrosome-associated Arp2/3 might be minor in adherent cells as compared with lymphocytes, given that they use this complex to form adhesive structures such as lamellipodia. Interestingly, it has been shown that immune synapses share many features with lamellipodia^40^, suggesting that the competition between the centrosome and the immune synapse for Arp2/3 might also apply to the centrosome and the lamellipodium of migrating cells.

In conclusion, our results highlight an unexpected role for the regulation of centrosome-associated F-actin in the control of lymphocyte polarization. Whether and how this novel regulatory mechanism applies to additional biological systems that rely on cell polarity is an open question. This would be of particular interest in the context of cilium biogenesis that involves signalling pathways also used in immune synapse formation by lymphocytes^41^,^42^.

## Methods

### Cells and cell culture

The mouse IgG^+^ B-lymphoma cell line IIA1.6 (derived from the A20 cell line (American Type Culture Collection #: TIB-208)) and the LMR7.5 T-cell hybridoma that recognizes I-A^d^-Lack_156–173_ complexes (from and described in ref. 43) were cultured as reported^14^ in CLICK medium (RPMI 1640—GlutaMax-I supplemented with 10% fetal calf serum, 1% penicillin–streptomycin, 0.1% β-mercaptoethanol and 2% sodium pyruvate). All cell culture products were purchased from GIBCO/Life Technologies. All experiments were conducted in 50% CLICK/50% RPMI 1640—GlutaMax-I.

### siRNA

Protein silencing was achieved using the Neon transfection system (Invitrogen, Life Technologies). Briefly, B cells were washed in phosphate-buffered saline (PBS), resuspended in Buffer R at a density of 50 × 10^6^ cells per ml and ON-TARGETplus SMARTpool Non-targeting (siCtrl), HS1- (siRNA#1: 5′-GGGCAUGAUGUAUCGGUUU-3′; siRNA#2: 5′-CCAAGGAGAGGGAAGCGAU-3′; siRNA#3: 5′-UGGAAGAGCCAGUGUACGA-3′; and siRNA#4: 5′-GUAAAGAUGAGCCGAGAAG-3′), Arp2- (siRNA#1: 5′-UGGUGUAACUGUUCGAUAA-3′; siRNA#2: 5′-GUUCUUUACUAAUGACGAA-3′; siRNA#3: 5′-GAUCAGUGCUUCUCGACAA-3′; and siRNA#4: 5′-CAUCGAGGUUGGAACGAGA), Arp3- (siRNA#1: 5′-GAAGAGAGCUAAGACGAUU-3′; siRNA#2: 5′-AAGCAGUGAAGGAACGCUA-3′; siRNA#3: 5′-GCUGACGGGUACAGUAAUA; siRNA#4: 5′-GAGUCAACGCCAUCUCAAA) or WASH- (siRNA#1: 5′-ACAGCAACACGGCGGAAUA-3′; siRNA#2: 5′-GAGGAGAAAUUGUUCGAUG-3′; siRNA#3: 5′-GCACAUUCAGGAACGUUUA-3′; and siRNA#4: 5′-GAAUACGGCUCCAUCUUUA-3′) targeting siRNA (Dharmacon, GE Healthcare) were added at a final concentration of 200 nM. Cells were then electroporated (1,300 V, 2 pulses, 20 ms per pulse) using 10-μl tips, and incubated in CLICK medium for 60–72 h. Silencing efficiency was analysed by western blot as described below.

### Plasmids

The eGFP–Centrin1 and Utrophin-RFP plasmids were obtained from M. Bornens and M. Piel, respectively (Institut Curie, Paris, France). The eGFP–Centrin1–VCA plasmid was obtained by sub-cloning in frame the VCA domain of WASH at the C terminus of the eGFP–Centrin1 construct. Briefly, the Centrin1 cDNA deleted from its stop codon was amplified from the eGFP–Centrin1 plasmid using the following primers: forward: 5′-ctaggtaccatggcttccggcttcaaga-3′ and reverse: 5′-gcaggatccgtaaaggctggtcttcttcat-3′ (which include KpnI and BamHI restriction sites (underlined), respectively). The WASH VCA cDNA was amplified from a previously home-made (A. Gautreau) WASH plasmid by using the primers: forward: 5′-ttaggatccTCCGGACTCAGATCTCGAGCTCAAGCTTCGAATTCTGCAGTCGACcagggagcccctaagga-3′ (which includes BamHI restriction site (underlined) and the sequence for a peptide linker (upper case)) and reverse: 5′-gattctagaTCAggactcccagtcctcct-3′ (which includes XbaI restriction site (underlined) and a stop codon (upper case)). Both fragments were then sub-cloned in frame within the original vector using KpnI and XbaI restriction enzymes. BamHI restriction enzyme was used to orient the two fragments. The resulting eGFP–Centrin1–VCA plasmid was sequenced to ensure sequence integrity. The LINC-DN (Nesprin-2 SRKASH) plasmid was obtained from E.R. Gomes and described in ref. 32. Plasmid expression was achieved by electroporating 2 × 10^6^ B-lymphoma cells, with 1 or 3 μg of plasmid using the Amaxa Cell Line Nucleofactor Kit R (T-016 programme, Lonza). Cells were incubated in CLICK medium for 16–20 h before analysis.

### Reagents

The Lack antigen was produced and purified by the ‘Recombinant Protein' platform (UMR144, Institut Curie, Paris, France), and Lack peptide (aa 156–173) was synthetized by PolyPeptide Group. Cytochalasin D and nocodazole used from centrosome purification were from Sigma Aldrich. For inhibition of Arp2/3 activity, cells were pretreated with 25 μM CK666 (Tocris Bioscience) for 30 min before being stimulated for indicated time in presence of the drug.

### Antibodies

The following primary antibodies were used for immunofluorescence: rabbit anti-γ-tubulin (Abcam, #Ab11317, 1/2000); fluorescein isothiocyanate (FITC)-conjugated mouse anti-α-tubulin (Abcam, #Ab64503, 1/100); rabbit anti-Arp2 (Abcam, #Ab47654, 1/200); rabbit anti-HS1 and rabbit anti-phospho-HS1 (both from Cell Signalling Technologies, #4557S and #8714P, respectively, 1/50); rabbit anti-WASH (home-made as previously described^44^, 1/250); and human anti-green fluorescent protein (GFP) and anti-red fluorescent protein (RFP) (Recombinant Antibodies Platform, Institut Curie, Paris, France, 1/200). The following secondary antibodies were used: AlexaFluor488-, AlexaFluor568-, Cy3-, Cy5- and AlexaFluor647-conjugated F(ab′)_2_ donkey anti-rabbit (Jackson ImmunoResearch, 1/300); and AlexaFluor488- and Cy3- conjugated donkey anti-human (Life Technologies and Jackson ImmunoResearch, respectively, 1/200). F-actin was stained using AlexaFluor546- or AlexaFluor647-conjugated phalloidin (Life Technologies, #A22283 and #A22287, respectively, 1/100). Nuclei were stained using 4′,6-diamidino-2-phenyindole (DAPI, Sigma Aldrich, 1/5,000).

For western blotting, the following antibodies were used: mouse anti-γ-tubulin (Sigma Aldrich, #T6557-.2ML, 1/500); rabbit anti-HS1, phospho-HS1 and phospho-Erk (Cell Signaling Technologies, #4557S, #8714P and #4377S, respectively, 1/1,000); rabbit anti-Arp2 (Abcam, #Ab47654, 1/200); rabbit anti-WASH (home-made as previously described^44^, 1/250); and mouse anti-Arp3 and anti-vinculin (Sigma Aldrich, #A5979-200UL, 1/200, and #V9264-200UL, 1/1,000, respectively), followed by horseradish peroxidase-conjugated donkey anti-mouse or rabbit (Jackson ImmunoResearch, 1/5,000).

### Preparation of BCR-ligand-coated beads

In all, 4 × 10^7^ 3-μm latex NH_2_-beads (Polyscience) were activated with 8% glutaraldehyde (Sigma Aldrich) for 2 h at room temperature. Beads were washed with PBS 1x and incubated overnight at 4 °C with different ligands: 100 μg ml^−1^ of either F(ab′)_2_ goat anti-mouse IgG (BCR-ligand^+^ beads) or F(ab′)_2_ goat anti-mouse IgM (BCR-ligand^−^ beads; MP Biomedical) in combination or not with 100 μg ml^−1^ of the *Leishmania major* antigen Lack.

### B-cell stimulation and immunofluorescences

Cells were plated on poly-L-lysine-coated slides and stimulated with indicated beads at a 1:2 ratio (cell:beads) for different time at 37 °C and fixed in 4% paraformaldehyde for 12 min at room temperature. For γ-tubulin staining, cells were further incubated with ice-cold 100% methanol for 2 min and quenched for 10 min with PBS/100 mM glycine. Fixed cells were incubated 45–60 min with primary antibodies and 30 min with secondary antibodies in PBS–BSA–Saponin (1x/0.2%/0.05%).

### Time-lapse imaging

A total of 1 × 10^5^ B-lymphoma cells co-expressing the centrosomal marker eGFP–Centrin1 and the F-actin probe Utrophin-RFP were seeded in 35-mm FD35 Fluorodish (World Precision Instruments, Inc). Cells were either treated with dimethylsulfoxide (DMSO) or CK666 (25 μM) or stimulated with either BCR-ligand^−^ or BCR-ligand^+^ beads and recorded at 37 °C, 5% CO_2_ using an inverted spinning disk confocal microscope (Roper/Nikon) equipped with a × 60 (1.4 numerical aperture (NA)) oil immersion objective and a CoolSNAP HQ2 camera. The images were acquired every 5 min with *z*-stack of 1 μm. For the analysis, Fiji (ImageJ) software was used to reconstruct the three-dimensional (3D) movies, correct bleaching (exponential fit correction) and analyse the amount of F-actin associated with the centrosome and the synapse focal plane.

### Stable isotope labelling by amino acids in cell culture

IIA1.6 cells were maintained in L-lysine-depleted SILAC RPMI 1640 (Thermo Scientific, Life Technologies) supplemented with 10% dialysed FBS and 0.1 mg ml^−1^ heavy [^13^C_6_] or light [^12^C_6_] L-lysine (Thermo Scientific, Life Technologies). Every 3–4 days, cells were split and media replaced with the corresponding light- or heavy-labelling medium. After six to seven cell divisions, cells achieved ≥96% incorporation of amino-acid isotopes.

### Centrosome purification

Centrosomes were purified as previously described^45^ with slight modifications. Briefly, following stimulation with indicated beads for 60 min, cells were incubated on ice with 200 nM nocodazole and 1 μg ml^−1^ cytochalasin D for 90 min. Cells were washed and lysed in TicTac buffer (16 mM PIPES, 10 mM HEPES (pH 7.5), 50 mM KCl, 1.2 mM EGTA, 5 mM MgCl_2_, 0.1% Triton X-100 and 0.1% β-mercaptoethanol) for 15 min. Centrosomes were isolated by sequential centrifugations at (1) 10,000*g* for 30 min at 4 °C on top of a 60% w/v sucrose cushion and (2) 40,000*g* for 60 min at 4 °C on top of a discontinuous sucrose gradient (40–50–70%, w/w). Finally, 10 fractions of 0.5 ml were recovered from the bottom of the tube, and centrosome-containing fractions were identified by western blot.

### Proteomics

*Sample preparation*. Proteins from centrosome preparations were separated on 10% SDS–PAGE gels (Invitrogen) and stained with colloidal blue staining (LabSafe GEL BlueTM GBiosciences). Gel slices were excised (20 fractions) and proteins were reduced with 10 mM dithiothreitol before alkylation with 55 mM iodoacetamide. After washing and shrinking of the gel fractions with 100% MeCN, in-gel digestion was performed using recombinant endoproteinase rLys-C (Promega) overnight in 25 mM NH_4_HCO_3_ at 30 °C.

*MS analysis*. Peptides were extracted and analysed by nano-LC–MS/MS using an Ultimate 3000 system (Dionex S.A.) coupled to a LTQ-Orbitrap XL mass spectrometer (Thermo Fisher Scientific), as described^46^. Samples were loaded on a C18 pre-column (300 μm inner diameter × 5 mm; Dionex) at 20 μl min^−1^ in 5% MeCN and 0.1% TFA. After 3 min of desalting, the pre-column was switched on the C18 column (75 μm inner diameter × 15 or 50 cm, packed with C18 PepMap, 3 μm, 100 Å; LC Packings) equilibrated in solvent A (5% CH_3_CN and 0.1% HCOOH). Bound peptides were eluted using a 97-min linear gradient (from 5 to 30% (v/v)) of solvent B (80% CH_3_CN and 0.085% HCOOH) at a 150-nl min^−1^ flow rate and oven temperature of 40 °C. Data-dependent acquisition was performed on the LTQ-Orbitrap mass spectrometer in the positive-ion mode. Survey MS scans were acquired in the Orbitrap on the 480–1,200 *m*/*z* range with the resolution set to a value of 60,000. Each scan was recalibrated in real time by co-injecting an internal standard from ambient air into the C-trap (lock mass option). The five most intense ions per survey scan were selected for collision-induced dissociation (CID) fragmentation and the resulting fragments were analysed in the linear trap (LTQ). Target ions already selected for MS/MS were dynamically excluded for 180 s.

*Data analysis*. Data were acquired using the Xcalibur software (v2.0.7) and the resulting spectra were analysed via the Mascot Software (v2.3) with Proteome Discoverer (v1.2, Thermo Scientific) using the SwissProt *Mus musculus* database. Carbamidomethylation of cysteine, oxidation of methionine, N-terminal acetylation and heavy ^13^C_6_-lysine (Lys6) were set as variable modifications. We set specificity of trypsin digestion and allowed two missed cleavage sites and mass tolerances in MS, and MS/MS were set to 2 p.p.m. and 0.8 Da, respectively. The resulting Mascot result files were further processed using *my*ProMS^47^ (v3.0), allowing a maximum FDR of 1% by automatically filtering the Mascot score at the peptide level.

*Protein quantification*. For SILAC-based protein quantification, peptides XICs (extracted ion chromatograms) were retrieved from Proteome Discoverer. Scale normalization computed using the ‘package limma' from R was applied to compensate for mixing errors of the different SILAC cultures as described^48^. Protein ratios were computed as the geometrical mean of related peptides. To estimate ratio significance, a *t*-test was performed with a Benjamini–Hochberg FDR control threshold set to 0.05. All quantified proteins have at least three peptides quantified (all peptides selected). Peptide intensity ratio outliers were removed when their value was too far from the median observed in the peptide intensity ratio set for a given protein. Protein quantification ratio outliers were not computed when the identified peptide number was too different between the two channels. Proteins displaying a minimal absolute fold change ≥10% that reaches statistical significance (adjusted *P* value of quantification ≤0.05) were considered as differentially associated with the centrosome of activated lymphocytes. This led to the selection of 835 proteins.

*GO term enrichment analysis*. Protein analysis by GO term enrichment was computed based on annotation only and did not take into account the relative abundance of the 835 proteins in resting and activated lymphocytes. The frequency of each GO was computed in the *Mus musculus* proteome (defined as the background, Slim Ontology file including all 21,283 mouse proteins) and compared with the set. We reported only the GO terms with the frequency statistically enriched in our protein set compared with the background. GO enrichment factors were computed with the GO::TermFinder^49^ through *my*ProMS. Briefly, to determine whether any GO term annotates a specified list of proteins at a frequency greater than the one expected by chance, GO::TermFinder calculates a *P* value using a hyper-geometric distribution. For multiple testing corrections, FDR was controlled and set to 1% (Benjamini–Hochberg). A *P* value was associated to each GO term individually. The FDR corresponds to the cutoff applied to the list of all the GO terms.

### *In vitro* nucleation assays

*In vitro* nucleation assays were performed according to Farina *et al*.^26^. In brief, centrosomes isolated in TicTac buffer were incubated for 20 min on surface of a polydimethylsiloxane open chamber. The excess of centrosomes was washed with TicTac buffer supplemented with 1% BSA (TicTac-BSA). Microtubule and actin nucleation was induced by diluting tubulin dimers (labelled with ATTO-565, 30 μM final^26^) or actin monomers (labelled with Alexa-568, 1 μM final^50^,^51^,^52^) in TicTac buffer supplemented with 1 mM GTP, 2.7 mM ATP, 10 mM dithiothreitol, 20 μg ml^−1^ catalase, 3 mg ml^−1^ glucose, 100 μg ml^−1^ glucose oxidase and 0.25% w/v methylcellulose. In addition, a threefold molar equivalent of profilin to actin was added in the reaction mixture^53^. Time-lapse observations were performed by using a total internal reflection fluorescence microscope (Roper Scientific) equipped by an iLasPulsed system and an Evolve camera (EMCCD 512 × 512, pixel=16 μm) using a × 60 1.49 NA objective lens. For quantification of the actin nucleation activity by centrosomes, F-actin fluorescence intensity was integrated over a 2-μm-diameter circle around the centrosome and normalized with respect to initial intensity. γ-Tubulin staining, needed for the efficiency calculation, was performed at the end of the movie recording under the microscope without prior fixation. Primary and secondary antibodies, diluted in TicTac-BSA buffer, were incubated for 60 and 30 min, respectively. Arp2/3 complex inhibition experiments were performed by adding 200 μM CK666 in reaction mixture. dimethylsulfoxide was used as control. Immunofluorescence staining of isolated centrosomes was performed by incubating centrosomes, seeded on a clean surface, with primary and secondary antibodies for 60 and 30 min, respectively.

### Western blotting

B cells were lysed at 4 °C in RIPA buffer (Thermo Scientific) supplemented with 1 × protease inhibitor cocktail (Roche) and 1 × Halt phosphatase inhibitor cocktail (Thermo Scientific). Supernatants were collected and loaded onto mini-PROTEAN TGX SDS–PAGE gels and transferred onto polyvinylidene fluoride membrane (Trans-Blot Turbo Transfer). Membranes were blocked in 5% non-fat dry milk resuspended in 1 × TBS–0.05% Tween-20 and incubated overnight at 4 °C, with primary antibodies followed by 60 min incubation with secondary antibodies. Western blots were developed with Clarity Western ECL substrate, and chemiluminescence was detected using the ChemiDoc imager (all from BioRad). Full scans of unprocessed western blots are available in Supplementary Fig. 7.

### Calcium measurement

A total of 1 × 10^6^ B cells were loaded with 1 μM Fluo-4 AM and Fura Red AM (Life Technologies) for 15 min at 37 °C in RPMI 1640 and resuspended in CLICK medium. The fluorescence of Fluo-4 and Fura Red were analysed using a BD Accuri C6 flow cytometer (BD Biosciences). After 120-s recording to assess basal Ca^2+^ levels, BCR-ligand at a final concentration of 10 μg ml^−1^ was added to the cell suspension and the Ca^2+^ levels were measured for 300 s. Finally, the ratio Fluo-4/Fura Red and the geometric mean over time were calculated using FlowJo software (v10, BD Biosciences).

### Antigen presentation

B cells were incubated with Lack±BCR-ligand-coated beads for 5 h or with peptide control for 1 h. Cells were washed with PBS, fixed in ice-cold PBS/0.01% glutaraldehyde for 1 min and quenched with PBS/100 mM glycine. B cells were then incubated with Lack-specific T-cell hybridoma in a 1:1 ratio for 24 h. Supernatants were collected and interleukin-2 cytokine production was assessed using BD optEIA Mouse IL-2 ELISA set following the manufacturer's instructions (BD Biosciences).

### Immunofluorescence acquisition and analysis

All *z*-stack images (0.5-μm spacing) were acquired on an inverted spinning disk confocal microscope (Roper/Nikon) with a × 60/1.4 NA oil immersion objective. Image processing was performed with Fiji (ImageJ) software^54^. Because methanol fixation required for γ-tubulin staining is not compatible with phalloidin labelling, the centrosome was stained with antibodies directed against α-tubulin, and a threshold was applied to visualize the centrosome but not the microtubules as described in Supplementary Fig. 2d. Single-cell images shown in the figures were cropped from large fields, rotated and their contrast and brightness manually adjusted. Images shown are the average *z*-projection of three planes around the centrosome.

Centrosome polarity index was computed as described in Fig. 1a. Briefly, *z*-stacks were projected (SUM slice) and images were automatically threshold (Default) on the green channel to obtain the centre of mass of the cell (Cell_CM_). Then, the position of the centrosome and the bead geometrical centre (Bead_GC_) were manually selected. The position of the centrosome was then projected (Cent_proj_) on the vector defined by the Cell_CM_–Bead_GC_ axis. The centrosome polarity index was calculated by dividing the distance between the Cell_CM_ and the Cent_proj_ by the distance between the Cell_CM_ and the Bead_GC_. The index ranges from −1 (anti-polarized) to 1 (fully polarized).

Centrosome- and synapse-associated Arp2 and F-actin were quantified as shown in Fig. 2b and Supplementary Fig. 4a, respectively. Briefly, after manual selection of the centrosome, background subtraction (rolling ball 50 px) on the *z*-projection (AVG) of the three planes around the centrosome was performed. We then computed the radial distribution of cytoplasmic Arp2 and F-actin fluorescence intensities from the centrosome of resting cells. The drop in fluorescence intensities (at 0.8 μm from the centrosome) was used as a threshold to define the radius of the centrosomal area that was further used to assess the amount of Arp2 and F-actin associated with the centrosome. The synaptic area was manually defined by positioning a fixed area at the cell–bead interface.

The distance between the centrosome and the nucleus was measured in three dimensions. For this, the nucleus was automatically threshold in 3D (Otsu) and the corresponding 3D-distance map was computed (Image 3D suite plugin). The 3D position of the centrosome was then manually selected on the cell stack and the shorter distance to the nucleus edge was measured on the 3D-distance map.

### Statistics

All graphs and statistical analysis were performed with GraphPad Prism 5 (GraphPad Software). No statistical method was used to predetermine sample size. Kolmogorov–Smirnov test was used to assess normality of all data sets. Mann–Whitney test was used to determine statistical significance excepted when mentioned. Boxes in box plots extend from the 25th to 75th percentile, with a line at the median and whiskers extend from the 10th to the 90th percentile. Bar graphs show the mean±s.e.m.

## Additional information

**How to cite this article**: Obino, D. *et al*. Actin nucleation at the centrosome controls lymphocyte polarity. *Nat. Commun.* 7:10969 doi: 10.1038/ncomms10969 (2016).

## Supplementary Material

Supplementary Information Supplementary Figures 1-7 and Supplementary Tables 1-5

Supplementary Movie 1 BCR stimulation reduces the ability of centrosome to nucleate F-actin. Time-lapse movie of F-actin assembly by centrosomes isolated from B cells stimulated with either BCR-ligand^-^ (left) or BCR-ligand^+^ (right) beads. Scale bar, 5 μm.

Supplementary Movie 2 Nucleation of F-actin at the centrosome relies on Arp2/3 activity. Resting B cells co-expressing the centrosomal marker eGFP-Centrin1 and the F-actin probe Utrophin-RFP were treated with DMSO (left) or CK666 (right) at indicated time (t_0_ min) and imaged by time-lapse spinning disk microscopy. The focal plane of the centrosome is shown. Scale bar, 3 μm.

Supplementary Movie 3 B cell stimulation decreases the pool of centrosomal F-actin. B cells co-expressing the centrosomal marker eGFP-Centrin1 and the F-actin probe Utrophin-RFP were stimulated with either BCR-ligand^-^ (top) or BCR-Ligand^+^ (bottom) beads at indicated time (t_0_ min) and imaged by time-lapse spinning disk microscopy. Cells shown in this movie are the same than the ones presented in Supplementary Movie 4 but the focal plane of the centrosome is shown. Scale bar, 3 μm.

Supplementary Movie 4 F-actin accumulates at the immune synapse upon B cell stimulation. B cells co-expressing the centrosomal marker eGFP-Centrin1 and the F-actin probe Utrophin-RFP were stimulated with either BCR-ligand^-^ (top) or BCR-Ligand^+^ (bottom) beads at indicated time (t_0_ min) and imaged by time-lapse spinning disk microscopy. Cells shown in this movie are the same than the ones presented in Supplementary Movie 3 but the focal plane of the synapse is shown. Scale bar, 3 μm.
