# Supplementary material for: Actin nucleation at the centrosome controls lymphocyte polarity
Source: Nat Commun. 2016 Mar 18;7:10969. doi: 10.1038/ncomms10969 (PMC4802043; doi:10.1038/ncomms10969)
Supplement: Supplementary Information — Supplementary Figures 1-7 and Supplementary Tables 1-5 [file ncomms10969-s1.pdf]

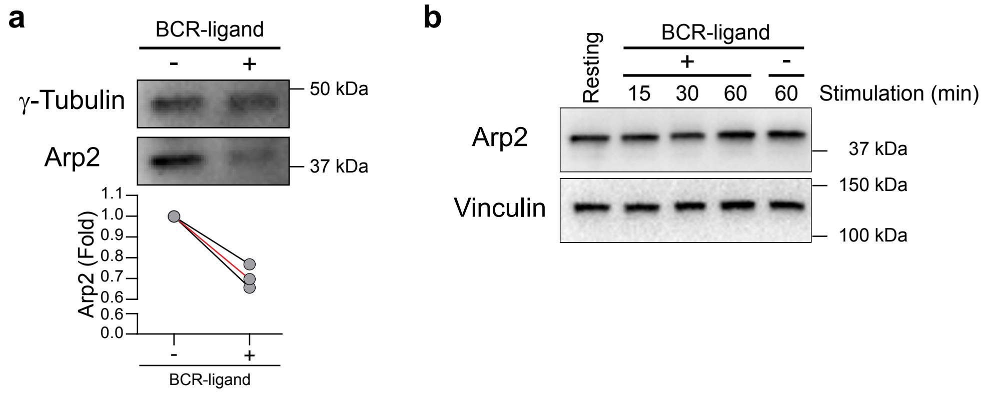

Supplementary Figure 1. Decrease association of Arp2/3 with the centrosome of BCR-stimulated lymphocytes. (**a**) Centrosomes purified from B cells stimulated with either BCR-ligand$^-$ or BCR-ligand$^+$ beads for 60 min were assessed by immunoblot for their associated amounts of Arp2. γ-Tubulin was used as loading control. Bottom panel shows the relative quantification of 3 independent experiments. Red line corresponds to the quantification of the blot presented above. (**b**) The total amount of Arp2 in B cells under resting conditions or stimulated with either BCR-ligand$^-$ or BCR-ligand$^+$ beads for indicated time was assessed by immunoblot. Vinculin was used as loading control. Western blots shown are representative of 3 independent experiments.

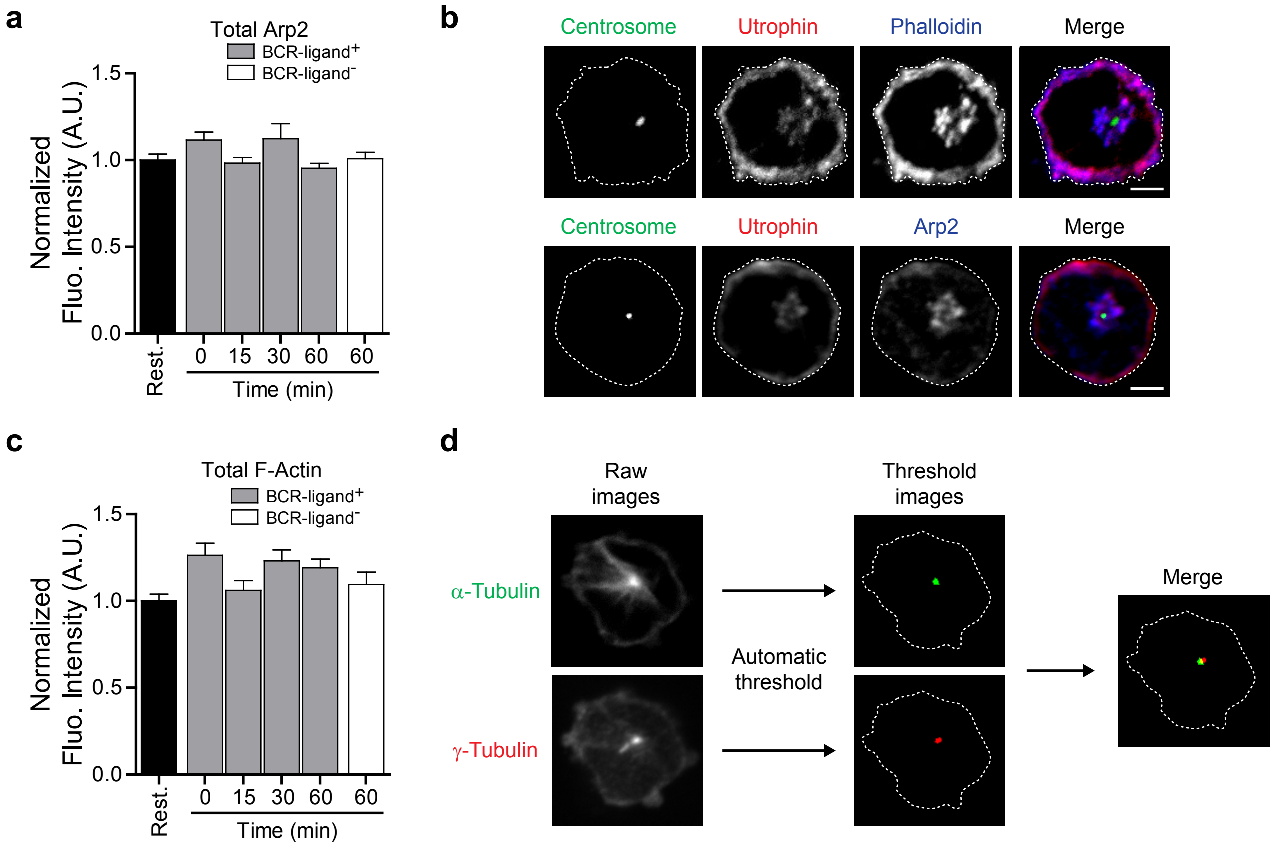

Supplementary Figure 2. Total levels of Arp2/3 and F-actin are not changed upon BCR-stimulation. (**a**) Quantification of the total Arp2 fluorescence intensity in B cells under resting conditions or stimulated with either BCR-ligand^{-} or BCR-ligand^{+} beads for indicated time. Data are pooled from 3 independent experiments and were normalized with respect to the mean fluorescence intensity of resting cells in each replicate. *n*=67, 71, 64, 68, 72 and 69 cells from left to right. (**b**) Representative images of resting B cells expressing the F-actin probe Utrophin-RFP, fixed and stained with either Phalloidin (top) or for Arp2 (bottom) and an anti-RFP (Utrophin). The merge images show the co-localization of Utrophin-RFP and Phalloidin staining (top) validating the use of Utrophin to label centrosomal F-actin as well as the co-localization of F-actin (Utrophin) and Arp2 at the centrosome (bottom). Images are representative of 2 independent experiments. Scale bar, 3 µm. (**c**) Quantification of the total F-actin fluorescence intensity in B cells under resting conditions or stimulated with either BCR-ligand^{-} or BCR-ligand^{+} beads for indicated time. Data are pooled from 3 independent experiments and were normalized with respect to the mean fluorescence intensity of resting cells in each replicate. *n*=60, 59, 64, 66, 59 and 64 cells from left to right. (**d**) Strategy used to threshold α-Tubulin staining to highlight the centrosome but not microtubules. The merge image shows the co-localization between α-Tubulin and γ-Tubulin staining validating the use of this approach to stain the centrosome. Images are representatives of 2 independent experiments.

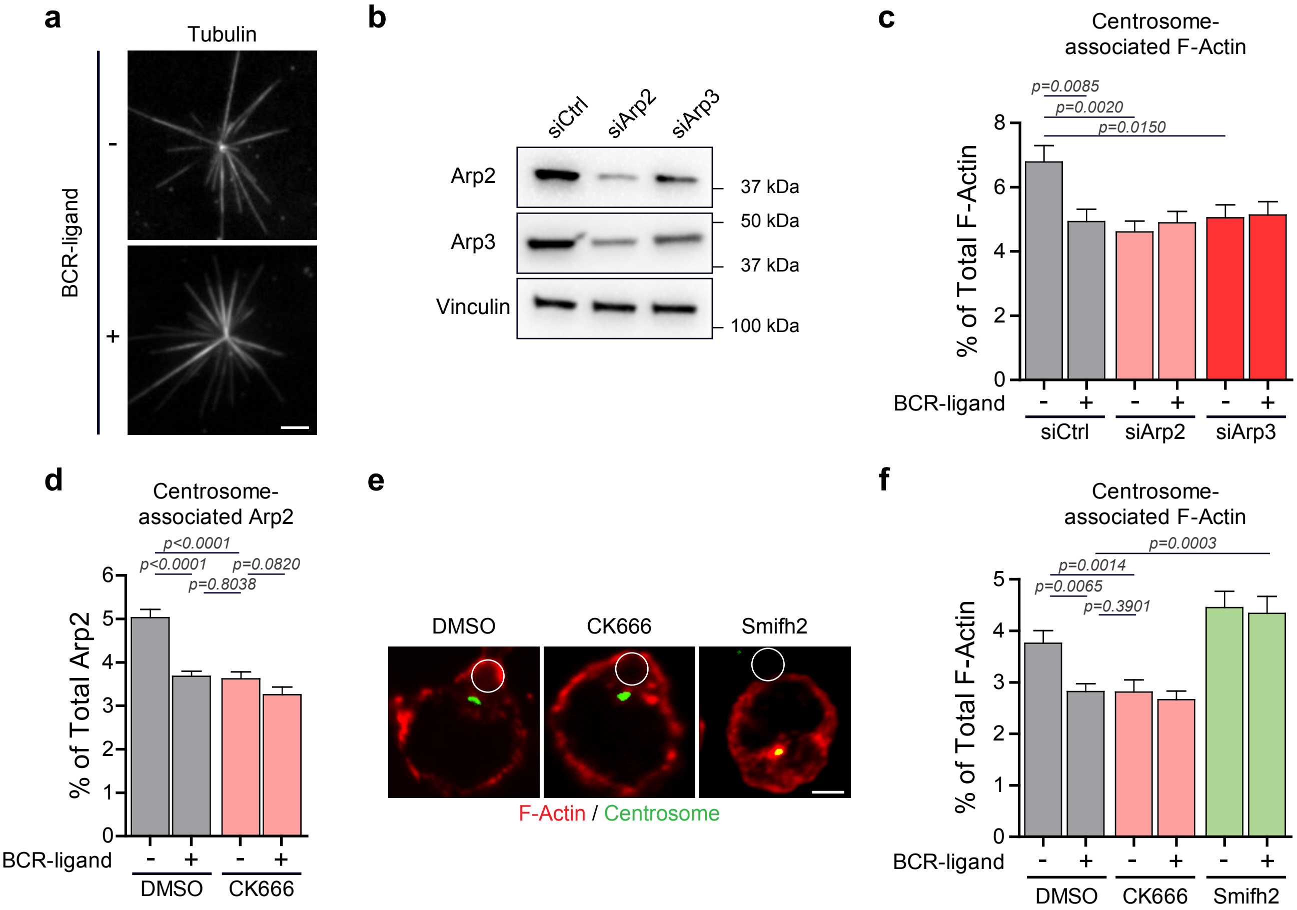

Supplementary Figure 3. F-actin nucleation by centrosomes is down-regulated upon BCR stimulation. (**a**) Centrosomes isolated from B cells stimulated for 60 min with indicated beads were assessed for their ability to nucleate microtubules *in vitro*. Scale bar, 16 μm. (**b**) Western blot analysis of the efficiency of Arp2 and Arp3 silencing. Vinculin was used as loading control. Immunoblot presented is representative of 2 independent experiments. (**c**) Quantification of centrosome-associated F-actin in control, Arp2- and Arp3-silenced B cells stimulated with indicated beads for 60 min. Data are pooled from 2 independent experiments with *n*=44, 45, 45, 46, 46 and 42 cells from left to right. (**d**) Quantification of centrosome-associated Arp2 in B cells treated with DMSO or CK666 and stimulated with indicated beads for 60 min. Data are pooled from 3 independent experiments with *n*=58, 62, 60 and 61 cells from left to right. (**e**) Representative images of B cells pre-treated with DMSO (vehicle control), CK666 (Arp2/3 inhibitor) or Smifh2 (Formin inhibitor) for 30 min prior to be stimulated with BCR-ligand^{+} beads for 60 min, fixed and stained for F-actin (Phalloidin) and the centrosome (α-Tubulin). White circles indicate bead position. Scale bar, 3 μm. (**f**) Quantification of centrosome-associated F-actin from cells shown in (e). Data are pooled from 3 independent experiments with *n*=82, 81, 68, 62, 76 and 69 cells from left to right. Data presented for DMSO and CK666 are the same than the one shown in Fig. 3h.

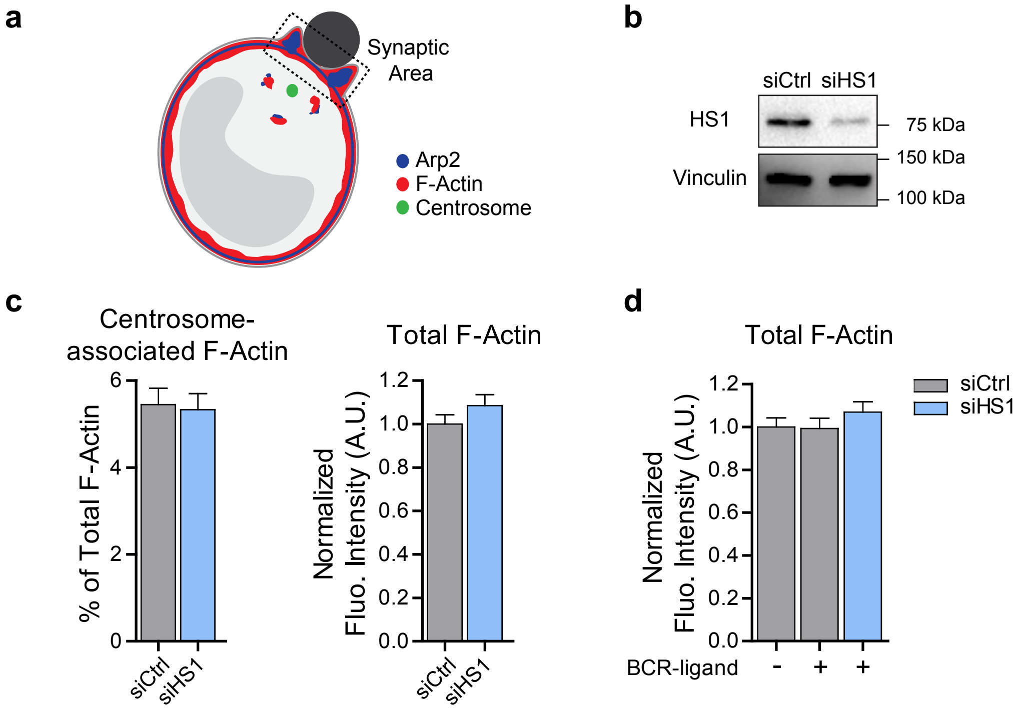

Supplementary Figure 4. Depletion of Arp2/3 from the centrosome results from its HS1-dependent recruitment at the immune synapse. (**a**) Schematics depicting the quantification of Arp2 and F-actin associated with the immune synapse. (**b**) Immunoblot analysis of the efficiency of HS1 silencing. Vinculin was used as loading control. The blot presented is representative of at least 4 independent experiments. (**c**) Quantification of centrosome-associated (left) and total (right) F-actin in control and HS1-silenced resting B cells. Data are pooled from 3 independent experiments with *n*=63 and 65 cells (left) and *n*=63 and 62 cells for siCtrl and siHS1, respectively. (**d**) Quantification of the total F-actin fluorescence intensity in control and HS1-silenced B cells stimulated with indicated beads for 60 min. Data are pooled from 3 independent experiments and were normalized with respect to the mean fluorescence intensity of control cells stimulated with BCR-ligand$^-$ beads in each replicate. *n*=72, 75 and 66 cells from left to right.

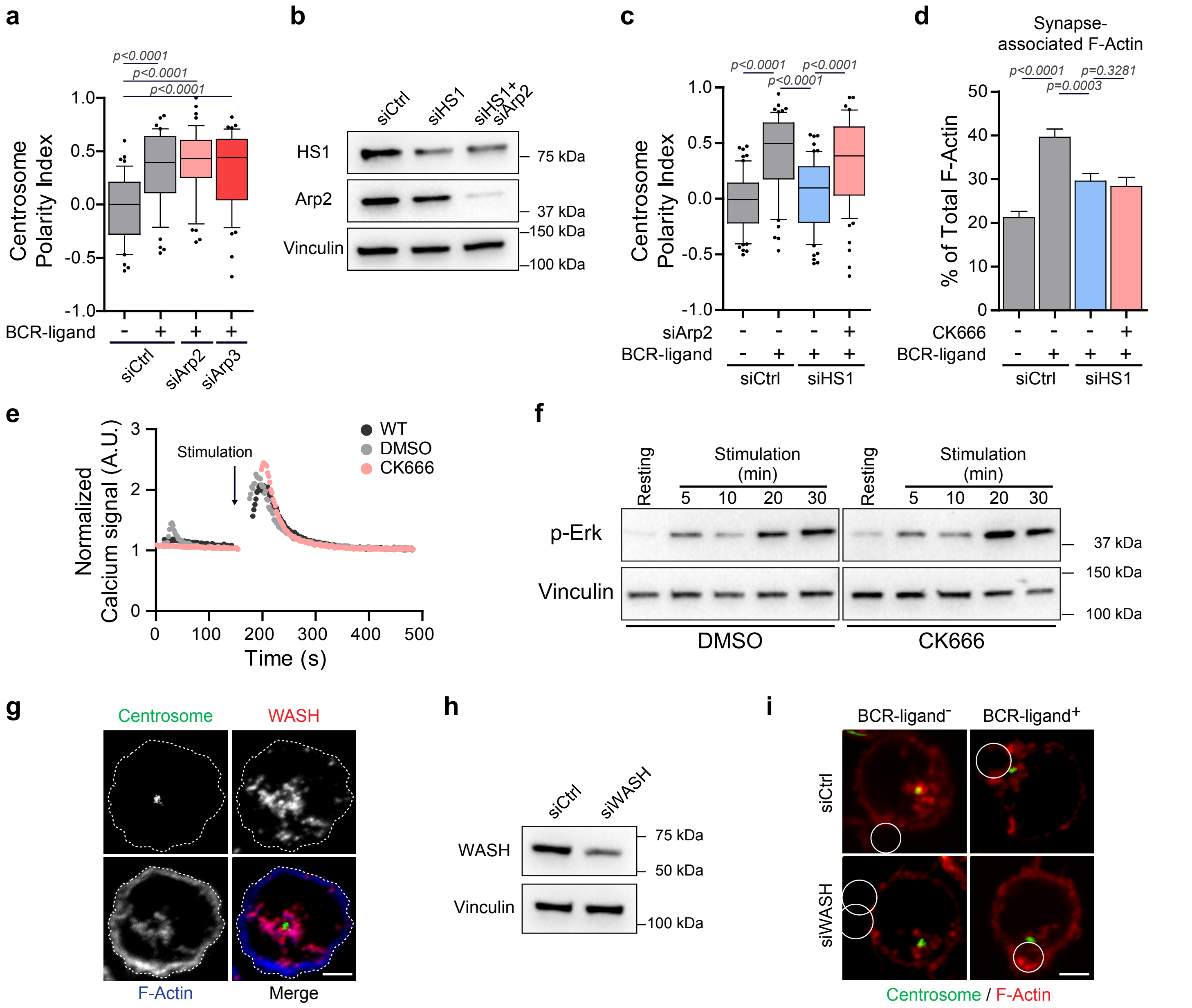

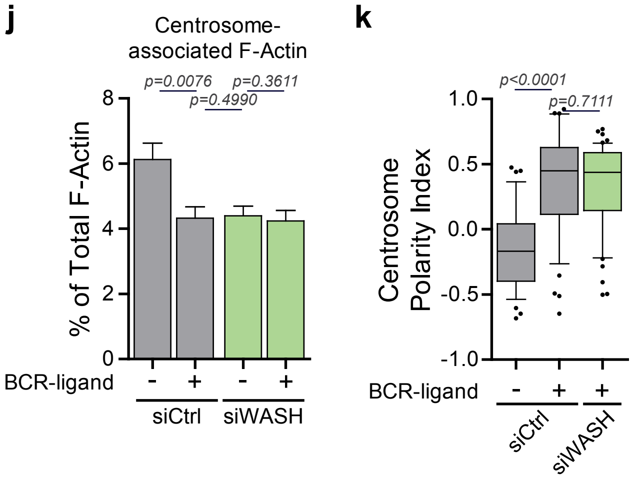

Supplementary Figure 5. Down-regulation of centrosomal Arp2/3-dependent F-actin nucleation is required for centrosome polarization to the immune synapse. (**a**) Quantification of centrosome polarity index in control, Arp2- and Arp3-silenced B cells stimulated with indicated beads for 60 min. Data are pooled from 2 independent experiments with *n*=43, 46, 47 and 43 cells from left to right. (**b**) Immunoblot analysis of the efficiency of HS1 and HS1 plus Arp2 silencing. Vinculin was used as loading control. Representative of 3 independent experiments. (**c**) Quantification of centrosome polarity index of control, HS1- and HS1 plus Arp2-silenced B cells stimulated for 60 min with indicated beads. Data are pooled from 3 independent experiments with *n*=64 cells per condition. (**d**) Quantification of synapse-associated F-actin of control and HS1-silenced B cells, treated with DMSO or CK666 and stimulated with indicated beads for 60 min. Data are pooled from 2 independent experiments with *n*=41, 47, 38 and 45 cells from left to right. (**e**) Intracellular calcium measurement of B cells treated or not with DMSO or CK666 and stimulated at indicated time (arrow) with soluble BCR ligand (10 µg.ml-1 final). Data are representative of 2 independent experiments performed in duplicate. Each dot represents the geometric mean. (**f**) Western blot analysis of phospho-Erk in B cells treated with DMSO or CK666. Immunoblots are representative of 3 independent experiments. (**g**) Representative images of resting B cells stained for WASH, F-actin (Phalloidin) and the centrosome (α-Tubulin). Images are representative of 2 independent experiments. Scale bar, 3 µm. (**h**) Immunoblot analysis of the efficiency of WASH silencing. Vinculin was used as loading control. Representative of 2 independent experiments. (**i**) Representative images of control and WASH-silenced B cells, stimulated with indicated beads for 60 min and stained for F-actin (Phalloidin) and the centrosome (α-Tubulin). White circles indicate bead position. Scale bar, 3 µm. (**j-k**) Quantification of centrosome-associated F-actin (**j**) and centrosome polarity index (**k**) in cells shown in (i). Data are pooled from 2 independent experiments with (**j**) *n*=36, 46, 41 and 50 cells and (**k**) *n*=37, 46 and 50 cells from left to right.

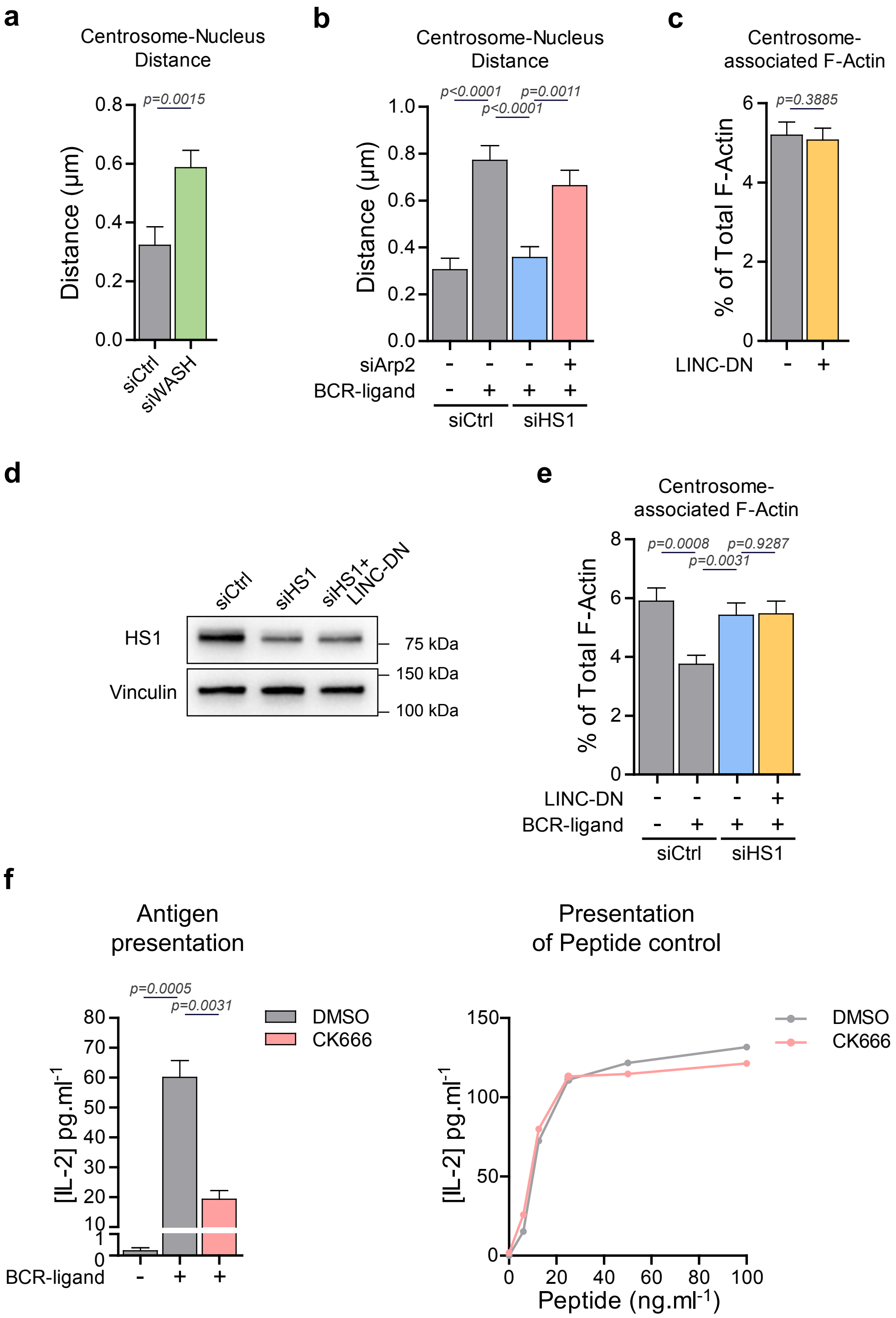

Supplementary Figure 6. Centrosomal Arp2/3-mediated F-actin nucleation links the centrosome to the nucleus through the LINC complex. (**a**-**b**) Quantification of the distance between the nucleus edge and the centrosome in: (**a**) control and WASH-silenced resting B cells and (**b**) control, HS1- and HS1 plus Arp2-silenced B cells stimulated for 60 min with indicated beads. Data are pooled from 2 (**a**) and 3 (**b**) independent experiments with (**a**) *n*=37 and 42 cells for siCtrl and siWASH, respectively and (**b**) *n*=64, 63, 61 and 64 cells from left to right. (**c**) Quantification of the amount of centrosome-associated F-actin in resting B cells over-expressing or not the LINC-DN construct. Data are pooled from 3 independent experiments with *n*=69 and 64 cells per condition. (**d**) Immunoblot analysis of the efficiency of HS1 silencing in cells over-expressing or not the LINC-DN construct. Vinculin was used as loading control. The blot presented is representative of 2 independent experiments. (**e**) Quantification of centrosome-associated F-actin in control and HS1-silenced B cells, over-expressing or not the LINC-DN construct and stimulated for 60 min with indicated beads. Data are pooled from 2 independent experiments with *n*=40, 37, 43 and 41 cells from left to right. (**f**) **Left.** Antigen presentation assay with control (DMSO) and CK666 treated B cells stimulated with indicated beads plus the Lack antigen. Bar graphs show the mean ± s.e.m. of triplicates. **Right.** Peptide control for cells used in the antigen presentation assay. Graph shows the mean of duplicates. Data are representative of 2 independent experiments.

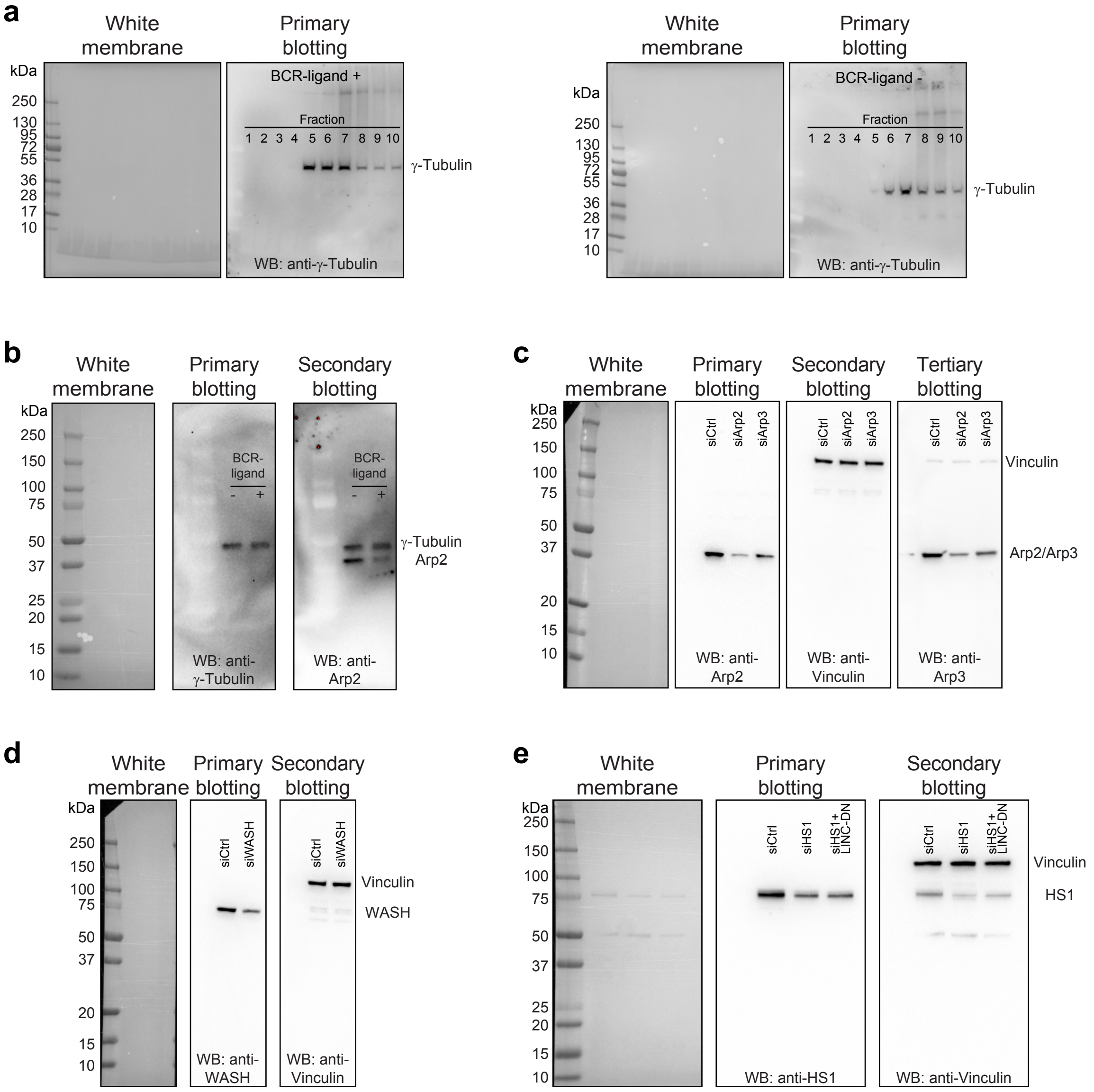

Supplementary Figure 7. Unprocessed scans of key Western blots. (**a**) Full scans of immunoblots shown in Fig. 1c. Membranes were sequentially incubated with an anti-γ-Tubulin antibody followed by the corresponding HRP-conjugated secondary antibody. (**b**) Full scans of immunoblots shown in Supplementary Fig. 1a. Blots were obtained as described in (a), except that the membrane was sequentially incubated with first an anti-γ-Tubulin antibody followed by the corresponding HRP-conjugated secondary antibody and second an anti-Arp2 antibody followed by the corresponding HRP-conjugated antibody. (**c**-**e**) Full scans of Western blots shown in Supplementary Fig. 3b (**c**), Supplementary Fig. 5h (**d**) and Supplementary Fig. 6d (**e**). Each blot was sequentially incubated with the indicated primary antibody and the corresponding HRP-conjugated secondary antibody. For loading controls, membranes were incubated sequentially with an anti-Vinculin antibody and revealed with the corresponding HRP-conjugated secondary antibody.

**GO Term** (cellular component)	**Number in Background (Total: 21283)**	**Frequency (%)**	**Number in Set (Total: 835)**	**Frequency (%)**	**Enrichment Factor**	***P-value***
Cell	16110	75.7	779	93.3	1,2	1,60E-42
Extracellular region	4228	19.9	214	25.6	1,3	2,12E-05
Intracellular	13050	61.3	775	92.8	1,5	1,09E-100
Cytoplasm	9739	45.8	589	70.5	1,5	2,20E-49
Organelle	11909	56.0	744	89.1	1,6	2,01E-100
**Cytoskeleton**	**1864**	**8.8**	**131**	**15.7**	**1,8**	**2,65E-11**
**Microtubule organizing center**	**607**	**2.9**	**45**	**5.4**	**1,9**	**3,56E-05**
Cytosol	1767	8.3	132	15.8	1,9	2,88E-13
Protein complex	4085	19.2	323	38.7	2,0	7,72E-41
Mitochondrion	1665	7.8	143	17.1	2,2	1,60E-19
Nucleus	6102	28.7	527	63.1	2,2	7,21E-99
Nuclear envelope	360	1.7	36	4.3	2,5	2,94E-07
Nucleoplasm	2356	11.1	272	32.6	2,9	1,73E-65
Nuclear chromosome	429	2.0	61	7.3	3,6	2,11E-18
Chromosome	784	3.7	115	13.8	3,7	1,85E-35
Peroxisome	132	0.6	21	2.5	4,1	4,62E-08
Nucleolus	814	3.8	152	18.2	4,8	2,13E-61
Ribosome	245	1.2	68	8.1	7,1	9,93E-39

Supplementary Table 1. List of Cellular Component enriched terms in proteins differentially associated with the centrosome of activated B cells. Table summarizing the Genome Ontology (GO) terms enriched in the set of the 835 proteins differentially associated with the centrosome of BCR-stimulated B cells. GO term enrichment factors were computed with GO::TermFinder with a FDR set to 1% (Benjamini-Hochberg) through myProMS software.

Centrosome / Microtubule	Activated/Resting			Peptide sets	
	Ratio	%	Adjusted *p-value*	Distinct	Used
CE170	1,23	23,03	4,49E-03	4	5
CHAP1	0,59	-41,48	4,06E-03	2	5
CHD4	0,65	-35,01	5,97E-16	5	19
CK5P3	1,31	30,90	2,56E-04	3	6
CKAP5	1,15	14,72	2,20E-33	31	108
CLAP1	1,14	14,09	3,62E-03	3	11
CLAP2	1,18	17,86	1,75E-05	3	13
CP110	1,29	28,67	3,07E-03	1	3
DCTN5	0,82	-18,25	2,81E-03	2	3
DLGP5	0,84	-15,53	3,67E-02	2	3
ECM29	1,22	21,77	6,57E-19	15	57
EMAL4	1,27	27,36	7,12E-07	6	16
EXOS7	0,89	-11,08	1,72E-03	3	5
GCP3	1,12	11,91	1,15E-03	5	10
GTSE1	1,53	52,51	4,66E-02	1	3
HAUS7	1,15	15,21	1,88E-02	2	3
IST1	0,80	-19,53	3,56E-03	2	6
JKIP1	1,23	22,70	3,78E-02	2	3
KAPCB	1,13	13,00	1,66E-02	2	3
KIF11	1,14	14,45	4,04E-12	18	70
KIF15	1,27	26,75	4,90E-13	12	31
KIF1C	1,31	31,03	3,02E-02	2	3
KIF4	0,66	-33,55	6,35E-03	2	4
KIFC1	0,73	-26,64	1,21E-03	1	6
KNTC1	1,25	25,49	2,44E-07	4	7
MACF1	1,10	10,02	1,57E-05	9	21
MAP4	1,13	12,69	1,08E-06	13	25
MAPK2	0,90	-9,77	3,20E-03	2	4
MARE1	0,73	-27,40	4,66E-03	2	3
MCM3	0,78	-21,69	1,85E-07	4	12
MDHC	1,24	24,44	4,34E-03	2	12
NEK7	1,11	11,28	1,71E-03	4	6
OFD1	1,24	24,47	8,43E-04	1	4
PCM1	1,19	19,28	8,81E-07	6	15
PLEC	0,87	-13,29	4,87E-06	16	16
RAGP1	0,86	-14,40	6,37E-11	15	54
RBBP6	0,70	-30,27	9,87E-03	3	5
RCC2	0,76	-23,71	2,71E-15	8	33
RGAP1	0,77	-22,66	4,23E-06	1	11
RTTN	1,24	23,97	3,94E-02	2	3
STIM1	1,20	19,63	2,30E-03	3	3
TBA4A	1,30	29,93	1,82E-04	2	10
TPX2	0,68	-32,46	4,06E-03	3	7
WDR11	1,12	11,95	3,40E-02	2	5
WDR43	0,67	-32,81	6,43E-05	3	6

Actin	Activated/Resting			Peptide sets	
	Ratio	%	Adjusted *p-value*	Distinct	Used
ACACA	1,20	20,03	9,63E-05	6	12
ACTB	0,83	-16,62	6,59E-13	4	25
ACTN4	0,85	-15,40	8,76E-10	7	24
ADDG	0,63	-37,45	2,54E-03	3	5
ARP2	0,88	-12,04	7,30E-10	6	42
ARP3	0,89	-10,66	5,44E-05	3	19
ARPC1B	0,90	-10,00	1,83E-09	5	37
CAPZB	0,83	-17,09	1,21E-06	5	14
CAZA1	0,84	-15,76	1,45E-05	4	7
CAZA2	0,84	-15,55	7,25E-03	4	4
CNN2	0,81	-18,76	1,41E-04	2	8
COF1	0,89	-11,30	6,70E-10	6	42
COR1C	0,83	-16,54	6,31E-07	2	14
DAAM1	0,83	-16,76	3,93E-02	4	5
DNM1L	0,83	-17,07	1,82E-08	11	48
DOCK2	1,13	13,36	2,55E-13	29	77
EM55	0,87	-13,01	2,67E-03	3	4
ENAH	0,87	-13,09	8,20E-05	2	6
EPB41	0,87	-12,69	1,76E-02	2	3
ERAL1	1,34	33,61	3,78E-02	1	5
FLNA	1,17	16,63	7,78E-13	19	34
FLNB	0,84	-16,33	6,46E-08	18	24
GBLP	1,18	18,21	4,72E-50	9	89
MERL	0,86	-13,51	6,81E-03	2	4
MRCKB	1,14	13,54	9,45E-03	3	8
MY18A	1,20	19,97	2,32E-15	22	47
MYH9	1,19	18,81	1,36E-39	53	202
PACN2	0,77	-23,00	2,54E-03	4	6
PDLI5	1,12	11,99	1,11E-03	3	10
PKN2	0,77	-23,02	4,82E-02	2	3
RAN	0,86	-14,14	1,09E-06	4	28
ROCK1	1,15	15,39	2,78E-04	4	10
ROCK2	1,15	14,53	1,61E-05	5	14
SPTB2	0,86	-13,58	2,33E-42	47	102
STML2	1,28	27,73	7,44E-12	5	21
TCRG1	0,70	-29,92	2,59E-04	4	5
TLN1	0,77	-22,73	1,38E-13	12	26
TLN2	0,57	-42,67	1,76E-09	11	14
TWF1	0,88	-11,53	1,47E-02	3	4
TWF2	0,84	-15,77	1,64E-03	4	5
URP2	0,90	-9,51	1,70E-02	2	6
VPS11	1,17	16,98	4,38E-02	4	13
WDR1	0,85	-15,36	6,22E-05	4	7
ACACA	1,20	20,03	9,63E-05	6	12
ACTB	0,83	-16,62	6,59E-13	4	25

Supplementary Table 2. Summary of SILAC-based protein quantification of microtubule and actin cytoskeleton components. To estimate ratio significance, a t-test was performed with a Benjamini–Hochberg FDR control threshold set to 0.05.

Sequence + Vmod	Charge	Individual ratio	Light	Heavy	RawFile source
VVVCDNGTGFVK + Carbamidomethyl (C:4)	2+	1/1.01	2.50e+06	2.53e+06	F6846FD.RAW
VVVCDNGTGFVK + Carbamidomethyl (C:4)	2+	1/1.07	2.11e+06	2.26e+06	F6848FD.RAW
VVVCDNGTGFVK + Carbamidomethyl (C:4)	2+	1/1.40	2.47e+06	3.46e+06	F6847FD.RAW
VVVCDNGTGFVK + Carbamidomethyl (C:4)	2+	1/1.09	1.37e+07	1.49e+07	F6853FD.RAW
VVVCDNGTGFVK + Carbamidomethyl (C:4)	2+	1/1.29	1.06e+06	1.36e+06	F6855FD.RAW
VVVCDNGTGFVK + Carbamidomethyl (C:4)	2+	1/1.22	1.02e+06	1.25e+06	F6852FD.RAW
VVVCDNGTGFVK + Carbamidomethyl (C:4)	2+	1/1.10	1.10e+06	1.21e+06	F6851FD.RAW
VVVCDNGTGFVK + Carbamidomethyl (C:4)	2+	1/1.16	6.84e+05	7.93e+05	F6850FD.RAW
VVVCDNGTGFVK + Carbamidomethyl (C:4)	2+	1/1.13	1.55e+06	1.75e+06	F6849FD.RAW
VVVCDNGTGFVK + Carbamidomethyl (C:4)	2+	1/1.07	6.32e+07	6.73e+07	F6854FD.RAW
VVVCDNGTGFVK + Carbamidomethyl (C:4)	2+	1/1.21	3.33e+05	4.02e+05	F6843FD.RAW
VVVCDNGTGFVK + Carbamidomethyl (C:4)	2+	1/1.29	2.08e+06	2.69e+06	F6845FD.RAW
ILLTEPPMNPTK	2+	1/1.19	4.12e+05	4.91e+05	F6851FD.RAW
ILLTEPPMNPTK	2+	1/1.07	1.23e+06	1.32e+06	F6855FD.RAW
ILLTEPPMNPTK	2+	1/1.11	3.16e+06	3.51e+06	F6844FD.RAW
ILLTEPPMNPTK	2+	1/1.05	1.26e+07	1.32e+07	F6854FD.RAW
ILLTEPPMNPTK	2+	1/1.13	1.45e+06	1.64e+06	F6848FD.RAW
ILLTEPPMNPTK	2+	1/1.10	4.26e+06	4.70e+06	F6845FD.RAW
ILLTEPPMNPTK	2+	1/1.14	1.89e+06	2.16e+06	F6847FD.RAW
ILLTEPPMNPTK	2+	1/1.24	6.57e+05	8.16e+05	F6843FD.RAW
ILLTEPPMNPTK	2+	1/1.10	2.11e+06	2.32e+06	F6846FD.RAW
ILLTEPPMNPTK	2+	1/1.07	7.42e+05	7.90e+05	F6850FD.RAW
ILLTEPPMNPTK + Oxidation (M:8)	2+	1/1.06	1.06e+06	1.12e+06	F6848FD.RAW
ILLTEPPMNPTK + Oxidation (M:8)	2+	1/1.13	2.73e+06	3.08e+06	F6844FD.RAW
ILLTEPPMNPTK + Oxidation (M:8)	2+	1/1.11	3.07e+05	3.41e+05	F6851FD.RAW
ILLTEPPMNPTKNREK	2+	1/1.09	1.03e+06	1.12e+06	F6854FD.RAW
LCYVGYNIEQEQK + Carbamidomethyl (C:2)	2+	1/1.18	2.21e+06	2.61e+06	F6844FD.RAW
LCYVGYNIEQEQK + Carbamidomethyl (C:2)	2+	1/1.03	1.54e+06	1.58e+06	F6847FD.RAW
LCYVGYNIEQEQK + Carbamidomethyl (C:2)	2+	1/1.28	4.72e+06	6.06e+06	F6854FD.RAW
LCYVGYNIEQEQK + Carbamidomethyl (C:2)	2+	1/1.07	9.55e+05	1.02e+06	F6846FD.RAW
LCYVGYNIEQEQK + Carbamidomethyl (C:2)	2+	1/1.43	6.48e+05	9.29e+05	F6848FD.RAW
LCYVGYNIEQEQK + Carbamidomethyl (C:2)	2+	1/1.48	3.16e+05	4.66e+05	F6850FD.RAW
LCYVGYNIEQEQK + Carbamidomethyl (C:2)	2+	1/1.08	2.97e+05	3.21e+05	F6856FD.RAW
LCYVGYNIEQEQK + Carbamidomethyl (C:2)	2+	1.05	8.53e+05	8.09e+05	F6855FD.RAW
QLYLERVLK	2+	1/1.10	4.17e+05	4.57e+05	F6851FD.RAW
QLYLERVLK	2+	1/1.18	1.97e+06	2.32e+06	F6845FD.RAW
QLYLERVLK	2+	1/1.00	4.50e+05	4.51e+05	F6855FD.RAW
QLYLERVLK	2+	1/1.09	1.14e+06	1.24e+06	F6848FD.RAW
QLYLERVLK	2+	1/1.17	1.99e+06	2.31e+06	F6844FD.RAW
QLYLERVLK	2+	1/1.12	1.54e+06	1.73e+06	F6847FD.RAW
QLYLERVLK	2+	1/1.03	1.03e+07	1.06e+07	F6854FD.RAW
QLYLERVLK	2+	1/1.15	9.66e+05	1.11e+06	F6846FD.RAW

Supplementary Table 3. Normalized individual peptide XICs identified for the ARP2 protein. Normalized individual peptide XICs (Extracted Ion Chromatograms) retrieved from Proteome Discoverer™ used to compute ARP2 protein ratio. For each peptide considered, the sequence and variable modification (Vmod) is provided as well as the charge, the individual ratio, the channel (light or heavy) intensity after normalization and the file name containing acquisition raw data (RawFile source).

Sequence + Vmod	Charge	Individual ratio	Light	Heavy	RawFile source
AGRLPACVVDCGTGYTK + Acetyl (Protein N-term) + Carbamidomethyl (C:7.11)	2+	1/1.23	3.01e+06	3.70e+06	F6844FD.RAW
AGRLPACVVDCGTGYTK + Acetyl (Protein N-term) + Carbamidomethyl (C:7.11)	2+	1/1.10	1.61e+05	1.78e+05	F6857FD.RAW
AGRLPACVVDCGTGYTK + Acetyl (Protein N-term) + Carbamidomethyl (C:7.11)	2+	1/1.07	2.22e+07	2.38e+07	F6852FD.RAW
AGRLPACVVDCGTGYTK + Acetyl (Protein N-term) + Carbamidomethyl (C:7.11)	2+	1/1.22	1.32e+06	1.61e+06	F6847FD.RAW
AGRLPACVVDCGTGYTK + Acetyl (Protein N-term) + Carbamidomethyl (C:7.11)	2+	1/1.12	3.03e+06	3.41e+06	F6850FD.RAW
AGRLPACVVDCGTGYTK + Acetyl (Protein N-term) + Carbamidomethyl (C:7.11)	2+	1/1.20	1.74e+06	2.10e+06	F6848FD.RAW
AGRLPACVVDCGTGYTK + Acetyl (Protein N-term) + Carbamidomethyl (C:7.11)	2+	1/1.05	2.69e+07	2.82e+07	F6853FD.RAW
AGRLPACVVDCGTGYTK + Acetyl (Protein N-term) + Carbamidomethyl (C:7.11)	2+	1/1.03	2.34e+06	2.42e+06	F6851FD.RAW
AGRLPACVVDCGTGYTK + Acetyl (Protein N-term) + Carbamidomethyl (C:7.11)	2+	1/1.09	2.34e+06	2.56e+06	F6849FD.RAW
AGRLPACVVDCGTGYTK + Acetyl (Protein N-term) + Carbamidomethyl (C:7.11)	2+	1/1.23	1.05e+06	1.30e+06	F6846FD.RAW
AGRLPACVVDCGTGYTK + Acetyl (Protein N-term) + Carbamidomethyl (C:7.11)	2+	1/1.23	5.24e+06	6.42e+06	F6845FD.RAW
AGRLPACVVDCGTGYTK + Acetyl (Protein N-term) + Carbamidomethyl (C:7.11)	2+	1/1.01	1.30e+05	1.32e+05	F6858FD.RAW
AGRLPACVVDCGTGYTK + Acetyl (Protein N-term) + Carbamidomethyl (C:7.11)	3+	1/1.11	1.46e+06	1.62e+06	F6853FD.RAW
YDTDGSKWIK	2+	1.01	5.02e+05	4.99e+05	F6851FD.RAW
YDTDGSKWIK	2+	1.01	2.98e+06	2.94e+06	F6852FD.RAW
QYTGVNAISK	2+	1/1.08	7.89e+06	8.49e+06	F6852FD.RAW
QYTGVNAISK	2+	1/1.10	4.91e+05	5.39e+05	F6848FD.RAW
QYTGVNAISK	2+	1/1.19	6.43e+05	7.65e+05	F6851FD.RAW
QYTGVNAISK	2+	1/1.29	2.19e+05	2.82e+05	F6849FD.RAW

Supplementary Table 4. Normalized individual peptide XICs identified for the ARP3 protein. Normalized individual peptide XICs (Extracted Ion Chromatograms) retrieved from Proteome Discoverer™ used to compute ARP3 protein ratio. For each peptide considered, the sequence and variable modification (Vmod) is provided as well as the charge, the individual ratio, the channel (light or heavy) intensity after normalization and the file name containing acquisition raw data (RawFile source).

Sequence + Vmod	Charge	Individual ratio	Light	Heavy	RawFile source
EVEERPAPTPWGSK	2+	1/1.02	2.45e+05	2.49e+05	F6855FD.RAW
ASSEGGAATGAGLDSLHK	2+	1/1.23	4.53e+05	5.55e+05	F6850FD.RAW
ASSEGGAATGAGLDSLHK	2+	1/1.12	6.52e+05	7.29e+05	F6843FD.RAW
ASSEGGAATGAGLDSLHK	2+	1/1.04	1.83e+06	1.90e+06	F6845FD.RAW
ASSEGGAATGAGLDSLHK	2+	1/1.21	4.90e+05	5.95e+05	F6856FD.RAW
ASSEGGAATGAGLDSLHK	2+	1/1.13	1.15e+06	1.29e+06	F6846FD.RAW
ASSEGGAATGAGLDSLHK	2+	1/1.08	2.31e+07	2.49e+07	F6855FD.RAW
ASSEGGAATGAGLDSLHK	2+	1/1.02	1.01e+07	1.03e+07	F6854FD.RAW
ASSEGGAATGAGLDSLHK	2+	1/1.08	1.50e+06	1.62e+06	F6847FD.RAW
ASSEGGAATGAGLDSLHK	2+	1/1.11	1.23e+06	1.36e+06	F6848FD.RAW
ASSEGGAATGAGLDSLHK	2+	1/1.04	2.69e+06	2.79e+06	F6844FD.RAW
ASSEGGAATGAGLDSLHK	2+	1/1.10	7.57e+05	8.34e+05	F6849FD.RAW
ASSEGGAATGAGLDSLHK	3+	1/1.04	1.85e+07	1.92e+07	F6855FD.RAW
ASSEGGAATGAGLDSLHK	3+	1/1.20	3.71e+05	4.44e+05	F6851FD.RAW
ASSEGGAATGAGLDSLHK	3+	1/1.25	4.71e+05	5.89e+05	F6843FD.RAW
ASSEGGAATGAGLDSLHK	3+	1/1.05	9.03e+05	9.48e+05	F6848FD.RAW
ASSEGGAATGAGLDSLHK	3+	1/1.05	1.10e+06	1.16e+06	F6846FD.RAW
NSVSQISVLSGGK	2+	1/1.10	6.70e+06	7.34e+06	F6845FD.RAW
NSVSQISVLSGGK	2+	1/1.11	3.82e+05	4.24e+05	F6805FD.RAW
NSVSQISVLSGGK	2+	1/1.01	2.03e+06	2.05e+06	F6856FD.RAW
NSVSQISVLSGGK	2+	1/1.01	1.82e+07	1.84e+07	F6854FD.RAW
NSVSQISVLSGGK	2+	1/1.15	2.65e+05	3.05e+05	F6852FD.RAW
NSVSQISVLSGGK	2+	1/1.32	1.52e+05	2.02e+05	F6804FD.RAW
NSVSQISVLSGGK	2+	1/1.22	1.37e+06	1.68e+06	F6843FD.RAW
NSVSQISVLSGGK	2+	1/1.09	4.14e+07	4.51e+07	F6855FD.RAW
NSVSQISVLSGGK	2+	1/1.17	3.56e+06	4.17e+06	F6848FD.RAW
NSVSQISVLSGGK	2+	1/1.15	6.16e+06	7.11e+06	F6844FD.RAW
NSVSQISVLSGGK	2+	1/1.17	1.02e+06	1.19e+06	F6846FD.RAW
NSVSQISVLSGGK	2+	1/1.16	1.19e+06	1.38e+06	F6849FD.RAW
NSVSQISVLSGGK	2+	1/1.04	3.34e+06	3.48e+06	F6847FD.RAW
NSVSQISVLSGGKAK	2+	1/1.16	8.04e+05	9.36e+05	F6849FD.RAW
NSVSQISVLSGGKAK	2+	1/1.07	4.41e+06	4.70e+06	F6854FD.RAW
NSVSQISVLSGGKAK	2+	1/1.22	2.17e+05	2.65e+05	F6852FD.RAW
NSVSQISVLSGGKAK	2+	1/1.09	4.10e+05	4.47e+05	F6855FD.RAW
NSVSQISVLSGGKAK	3+	1/1.03	2.10e+06	2.17e+06	F6854FD.RAW
NSVSQISVLSGGKAK	3+	1/1.09	7.38e+05	8.06e+05	F6849FD.RAW
SLESALKDLK	2+	1.00	5.74e+06	5.72e+06	F6855FD.RAW

Supplementary Table 5. Normalized individual peptide XICs identified for the ARPC1B protein. Normalized individual peptide XICs (Extracted Ion Chromatograms) retrieved from Proteome Discoverer^{TM} used to compute ARPC1B protein ratio. For each peptide considered, the sequence and variable modification (Vmod) is provided as well as the charge, the individual ratio, the channel (light or heavy) intensity after normalization and the file name containing acquisition raw data (RawFile source).
